# Retrospective chart review demonstrating effectiveness of bimodal neuromodulation for tinnitus treatment in a clinical setting

**DOI:** 10.1038/s43856-025-00837-3

**Published:** 2025-04-28

**Authors:** Emily E. McMahan, Hubert H. Lim

**Affiliations:** 1 Alaska Hearing & Tinnitus Center, Anchorage, AK USA; 2 College of Health, University of Alaska Anchorage, Anchorage, AK USA; 3 Department of Communication Sciences and Disorders, University of South Dakota, Vermillion, SD USA; 4 Department of Otolaryngology-Head & Neck Surgery, University of Minnesota, Minneapolis, MN USA; 5 Department of Biomedical Engineering, University of Minnesota, Minneapolis, MN USA; 6 Neuromod Devices Limited, Dublin, Ireland

**Keywords:** Neuropathic pain, Pain management

## Abstract

**Background:**

Bimodal neuromodulation combining sound therapy with electrical tongue stimulation using the Lenire® device is emerging as an effective treatment for tinnitus.

**Methods:**

A single-arm retrospective chart review of 220 patients with tinnitus from the Alaska Hearing & Tinnitus Center examines the real-world outcomes of the recently FDA-approved Lenire treatment. To the best of our knowledge, this is the first assessment of Lenire from a real-world clinic in the United States. The primary endpoint examines the responder rate and mean change in Tinnitus Handicap Inventory (THI) after ~12 weeks of treatment in eligible patients with moderate or worse tinnitus. A responder represents a THI improvement of greater than seven points (i.e., minimal clinically important difference, MCID).

**Results:**

Here we show that, out of 212 patients with available data, there was a high responder rate of 91.5% (95% CI: 86.9%, 94.5%) with a mean improvement of 27.8 ± 1.3 (SEM) points, and no device-related serious adverse events. Furthermore, a THI MCID of seven points represents a consistent criterion for clinical benefit based on evidence from clinical practice settings.

**Conclusions:**

These findings show that the Lenire device can be used to safely and effectively reduce tinnitus in a real-world clinical setting.

## Abstract (web summary)

McMahan and Lim evaluate the real-world effectiveness of Lenire, an FDA-approved bimodal neuromodulation device, in alleviating tinnitus in 220 patients attending a U.S. clinic. The findings corroborate results from previous clinical trials and further support Lenire as an effective treatment for tinnitus with a high responder rate of 91.5%.

## Plain language summary

Tinnitus is a condition where people hear sounds, like ringing, buzzing, or hissing, when no external sound is present. It affects 10–15% of the population and can cause substantial distress. Lenire is a device that has recently been approved by the FDA because it has been shown to reduce tinnitus in large research studies. It works by altering the way the brain experiences phantom sounds by providing sound stimuli to the ear and electrical stimuli to the tongue at the same time. We investigated whether it also reduced tinnitus in real world settings and found it reduced tinnitus symptoms. These results confirm that Lenire can be used as a treatment for tinnitus in a clinical setting.

## Introduction

Tinnitus, the phantom auditory experience, affects 10–15% of the global population^1–6^. For some, tinnitus is a minor annoyance while for approximately 6–11% of the tinnitus population, the experience is bothersome^3^. Treatments for bothersome tinnitus aim to alleviate tinnitus distress and help treatment-seeking individuals cope with the condition. These can include sound therapy, cognitive behavioral therapy (CBT), tinnitus retraining therapy (TRT), and newer neuromodulation treatments, among others^7–9^. When left unmanaged, bothersome tinnitus can be debilitating with a substantial negative impact on the patient’s quality of life^10^. Overall, tinnitus remains a major health issue in our society.

One promising non-invasive and accessible treatment approach supported by animal studies^11^ and several large-scale clinical trials is bimodal neuromodulation^12–14^, which combines sound therapy with electrical tongue stimulation using the self-administered, easy-to-use Lenire® device (Neuromod Devices, Ireland). More recently in March 2023, Lenire was granted De Novo approval for the treatment of tinnitus by the Food and Drug Administration (FDA; DEN210033)^12^. Results from the controlled pivotal clinical trial that was designed with guidance from the FDA confirmed that for those with moderate or more severe tinnitus symptoms (Tinnitus Handicap Inventory, THI, ≥38), when starting bimodal treatment, a clinically significant superior performance of bimodal neuromodulation (i.e., improvement greater than seven points on THI) was achieved with just 6 weeks of treatment compared to sound-only stimulation^12^. The 7-point improvement in THI is a criterion for the minimal clinically important difference (MCID) that has been estimated with a combination of an anchor-based method (i.e., CGI-I) and distribution-based method (i.e., effect size of 0.5 of SD)^15^, consistent with FDA guidelines for determining a threshold for significant change^16^.

Although the controlled pivotal trial led to positive results for tinnitus treatment, the critical question remains: how will Lenire perform in a less structured, real-world clinical setting with a more heterogeneous patient population than a clinical study? After FDA approval, 14 independently owned United States clinics began offering the device for tinnitus treatment commercially. Data from one of these clinics, the Alaska Hearing & Tinnitus Center, are included in this review. Patient data from the other 13 United States clinics are not included in this study as they are independently owned private practices and patient charts are not accessible by the Alaska Hearing & Tinnitus Center, which conducted this review. Furthermore, the Alaska Hearing & Tinnitus Center has currently treated the largest number of patients with the Lenire device and decided to complete a retrospective chart review to share their results. This single site, single arm, chart review documents the results of 220 patients fitted with Lenire between May 4, 2023 and March 28, 2024. At most clinics, tinnitus management conventionally involves in-person care; however, since the COVID-19 pandemic, telehealth has become instrumental for routine follow-ups and consultations^17^. Thus, at the Alaska Hearing & Tinnitus Center, a hybrid model was used to treat patients with tinnitus, where the Lenire device fitting was conducted in person, and follow-up appointments were performed virtually for most patients.

After initial consultation (in-person or via telehealth), if a patient was prescribed Lenire, an in-person device fitting was completed. During in-patient fitting, the intensity of the electrical tongue stimulus was calibrated to a comfortable sensation level for the patient and the sound stimulus was adjusted to a comfortable loudness based on the audiogram for each patient. All patients utilized the same stimulation setting, which presented pure tones to the ears while simultaneously presenting electrical pulses to the top surface of the tongue (further details provided in previous publications)^12,13,18^. Patients were provided with the user manual and comprehensive training with the device, including what to expect from the treatment, potential side effects, and how to use the device. Patients were instructed to use the device for up to 60 min per day for at least 12 weeks. Follow-on care and assessments were carried out approximately halfway through their treatment (FU1) and ~12 weeks (FU2) after fitting; these appointments were completed via telehealth with the option for in-person care for those who were located closer to a clinic. The online services facilitate continued care by providing the option for remote tinnitus counseling, education, and additional consultations.

In this retrospective chart review, THI is measured in 220 patients attending the Alaska Hearing & Tinnitus Center before and ~12 weeks after beginning Lenire treatment to determine its ability to reduce tinnitus in a real-world clinical setting. The results show that 91.5% of patients exhibit a clinically meaningful reduction in tinnitus in response to Lenire and 89.2% of patients report Lenire to be beneficial for their tinnitus journey.

## Methods

### Study design

**Fig. 1:**
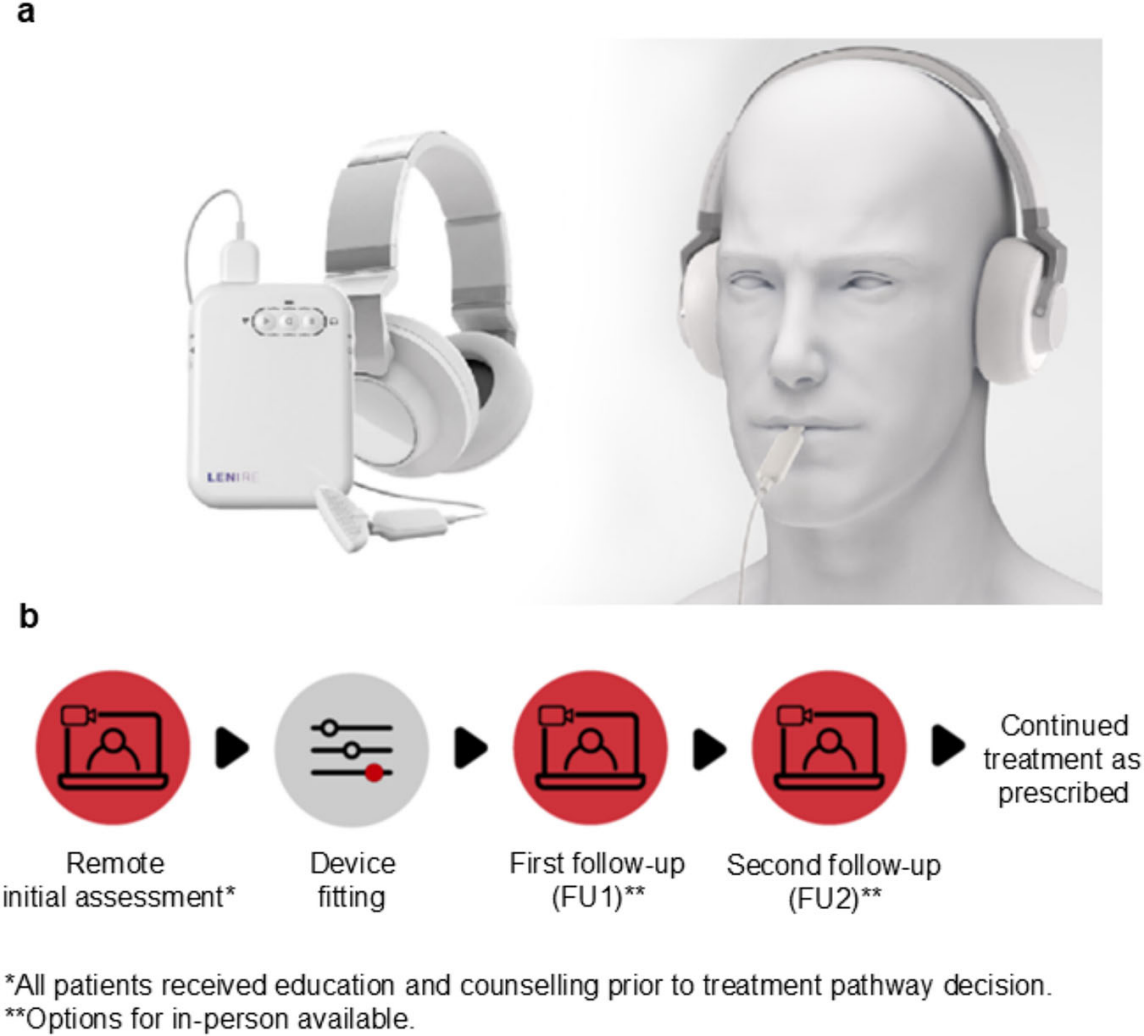
Bimodal neuromodulation treatment and study design. Image in **a** reprinted from “Boedts, M. et al. Combining sound with tongue stimulation for the treatment of tinnitus: a multi-site single-arm controlled pivotal trial. *Nature Communications* **15**, 6806 (2024).” **a** Lenire bimodal neuromodulation device by Neuromod Devices (Dublin, Ireland) intended to reduce the symptoms of tinnitus in patients with moderate or worse tinnitus (i.e., FDA-approved for those with a Tinnitus Handicap Inventory (THI) score ≥38). The device consists of a Tonguetip®, an intraoral device designed to sit comfortably in the mouth and deliver gentle electrical stimulation on the tongue’s surface; Bluetooth headphones that play personalized sounds to the ears; and a handheld controller for patients to adjust the duration and intensity of the treatment. The patient has a limited range of control over the sound volume and tongue stimulation with the handheld controller for ease of comfort and to maintain stimulation sensations at a noticeable but near-threshold level. **b** Lenire standard of care procedure at Alaska Hearing & Tinnitus Center.

This study is a single-site, single-arm retrospective analysis of 220 patients who were fitted with the Lenire device (Fig. 1a) from May 4, 2023 to March 28, 2024 at the Alaska Hearing & Tinnitus Center clinic. The patients were fitted based on the indications for use for Lenire in patients 18 years of age and older suffering from at least moderate tinnitus severity (THI ≥ 38 at initial assessment). This observational study was reviewed by a registered IRB (name: Advarra IRB; IRB number: 00000971; Study Protocol number: Pro00077817) and was determined to be exempt from IRB oversight based on the Department of Health and Human Services regulations found at 45 CFR 46.104(d)(4). All methods were carried out in accordance with relevant guidelines and regulations outlined in the study protocol, which is available in the Supplementary Methods.

Medical records stored in CounselEAR Office Management Solutions (OMS) were accessed by the clinician who had access to the records as part of her standard duties at the clinic. Upon exporting the data, no identifiable information was retained in the final study database, nor will there be a need to re-identify the data. Patients in this study will not be contacted and have given written consent to a HIPAA Waiver Notice (‘Notice of Privacy Practices’) prior to initial treatment at the clinic, which allows for their data to be used for research purposes. It was verbally explained during the initial assessment how the data would be used to further research on the Lenire device. As such, these patients gave informed consent for their data to be used in this study. A template of the clinic’s Privacy Policy and Notice of Privacy Practices was attached to the IRB submission as supporting information.

All patients in this study attended an initial assessment either via CounselEAR’s telehealth portal or in the clinic. This visit included health evaluations and several tinnitus assessments. All patients received tinnitus counseling and education before selecting a treatment plan that best supported their hearing and tinnitus needs. The audiologist reviewed appropriate expectations regarding outcomes and treatment compliance with each patient. For patients who attended their initial assessment via telehealth, a subsequent in-person appointment was scheduled for the fitting of the Lenire device. Patients who had their initial assessment in person could be fitted with the device during that initial assessment appointment if they wished and if time permitted, or during a separate in-person fitting appointment. During the fitting appointment, patients were provided with a device training session and the Lenire User Manual. The electrical tongue stimulus intensity was calibrated to a comfortable sensation level, and the sound stimulus was adjusted to a comfortable loudness based on the audiogram for each patient. They were instructed to use the device for up to 60 min per day for at least 12 weeks (Fig. 1b). Severity of tinnitus was assessed using the THI at FU1 approximately halfway through their treatment, and at a second follow-up ~12 weeks from initial fitting (FU2). Patients could contact the clinic at any time between appointments if there were any concerns. Virtual video calls were primarily used for follow-up assessments, additional consultations, counseling, and education. All devices were fitted and follow-up assessments were conducted by Emily E. McMahan, who is the audiologist at the Alaska Hearing & Tinnitus Center, ensuring consistency in the fitting process and patient care.

The Lenire neuromodulation system is an FDA-approved take-home device. Treatment sessions were self-administered by patients at home. A detailed description of the device has been published in previous Lenire clinical trials^12–14^. All patients in this study utilized the same stimulation setting (PS1), which includes pure tones presented to the ears that are synchronized with electrical pulses presented to the top surface of the tongue. There are currently two stimulation settings, PS1 and PS6, which are approved by the FDA for tinnitus treatment in the United States based on the results from the TENT-A3 pivotal trial and supporting clinical data included in the FDA De Novo submission. A full description of the PS1 and PS6 settings can be found in previous clinical trial publications^13,14^.

### Participants

Lenire is prescribed to patients 18 years and older with subjective tinnitus that is moderate or worse in terms of tinnitus severity (THI ≥ 38). The device is contraindicated, unless directed by a physician, or where applicable a dentist, for those who are pregnant; have an active implantable device; have conditions that cause impaired sensitivity in the tongue; have epilepsy or other conditions which may cause loss of consciousness; have sores, lesions, or inflammation of the oral cavity; have chronic or intermittent neuralgia in the head and neck area; have Meniere’s disease. Lenire is contraindicated for those who have tinnitus confirmed to be from an objective source; and those who cannot remove oral piercings during device use. Of the 220 patients who were fitted with Lenire at initial assessment, 41.4% (*n* = 91) self-reported as current hearing aid users. As part of the initial assessment questions, hearing aid users were asked if they had their hearing aid fitted in the past 90 days; if they indicated ‘Yes’, then they were not prescribed Lenire at that time.

### Clinical endpoints

One of the main objectives of this retrospective chart review is to assess real-world data to determine the replicability of Lenire treatment outcomes observed in previous clinical trials within a clinical practice setting. To the best of our knowledge, this is the first time evaluating the effectiveness of Lenire in a real-world clinical setting within the United States. Therefore, in line with previous clinical trials, THI was used at all stages to assess tinnitus symptom severity for comparability to existing published results^12–14^.

The THI is a validated 25-item questionnaire measuring perceived tinnitus handicap severity anchored with “No” (0 points), “Sometimes” (2 points), or “Yes” (4 points) responses^19^. These scores are added up to a total value ranging from 0 to 100, where a higher score indicates a higher level of tinnitus severity. The THI can also be divided into five severity categories: slight (0–16 points), mild (18–36 points), moderate (38–56 points), severe (58–76 points), and catastrophic (78–100 points). The MCID reported for THI is seven points and represents a clinically meaningful change in tinnitus symptoms^15^. It is noteworthy that while the THI is based on a three-category scoring system, which has been validated through response frequency distribution, item-total correlations, and content validity^19^, there are ongoing discussions within the scientific community about analyzing it as an ordinal response^20,21^. Since the three-category scoring system is still widely used in the tinnitus field and to be consistent with how it has been analyzed in prior Lenire clinical trials, a similar methodology was used at the Alaska Hearing & Tinnitus Center. This approach allows for direct comparison with previously published research, strengthening the validity of our findings and supporting the broader applicability of Lenire treatment outcomes in real-world clinical practice settings.

In addition to the THI, patients were asked at FU2 to respond ‘Yes’ or ‘No’ to the question: “Do you find Lenire beneficial to your tinnitus journey?”

### Field safety reporting

The Lenire device was approved for distribution in the United States in March of 2023 by the FDA. All healthcare professionals providing the Lenire device may submit information in relation to adverse events (AEs), technical issues, or general feedback directly to the manufacturer (Neuromod Devices) through the Zoho ticketing system. Once a ticket is received, it is assessed for alleged device malfunction, device fitting problems, undesired product performance, or other adverse field experiences (e.g., undesirable medical symptoms). Based on this assessment, a determination is made on whether a Field Product Experience Report (FPER) should be opened to further document and investigate the report. Once an FPER is opened, an initial determination of the need to submit a report to the FDA (in the United States) or Competent Authorities (outside the United States) is conducted. Reports are assessed by the clinical or technical teams at the manufacturing company, as appropriate, for the need for further information or follow-up. Where an FPER is determined to warrant reporting to the FDA or a Competent Authority, this is completed using the process and timeline for the applicable jurisdiction. There were no medical FPERs submitted to the manufacturer, and all technical queries were resolvable with no reporting actions required.

### Statistics and reproducibility

The primary endpoint consisted of a responder rate analysis where the responder rate was calculated as the percentage of participants achieving more than seven points reduction in THI from initial assessment to FU2. Responder rate is reported with corresponding 95% CIs. The primary endpoint also included the mean change in symptoms of tinnitus based on THI from initial assessment to FU2 that is reported with corresponding SEM. Additional analyses are reported for responder rate and mean change from initial assessment to FU1, as well as using a more conservative MCID criterion of 9 points based on new findings observed in this study.

To assess differences in performance between follow-up visits, a two-sided *Z*-test of proportions on responder rates and an independent t-test on mean changes were performed for the THI values. In addition, a paired t-test was conducted to assess the difference between follow-up visits in mean changes in THI for patients who returned for both follow-up visits. At the initial visit, participants self-reported if they were male or female. Analyses were further carried out according to participants’ self-reported sex. Of the 220 participants, 161 were male and 59 were female. All statistical analyses were conducted with Stata 15. Bonferroni corrections were applied to all statistical analyses involving multiple comparisons.

### Reporting summary

Further information on research design is available in the Nature Portfolio Reporting Summary linked to this article.

## Results

### Characteristics of patients

**Fig. 2:**
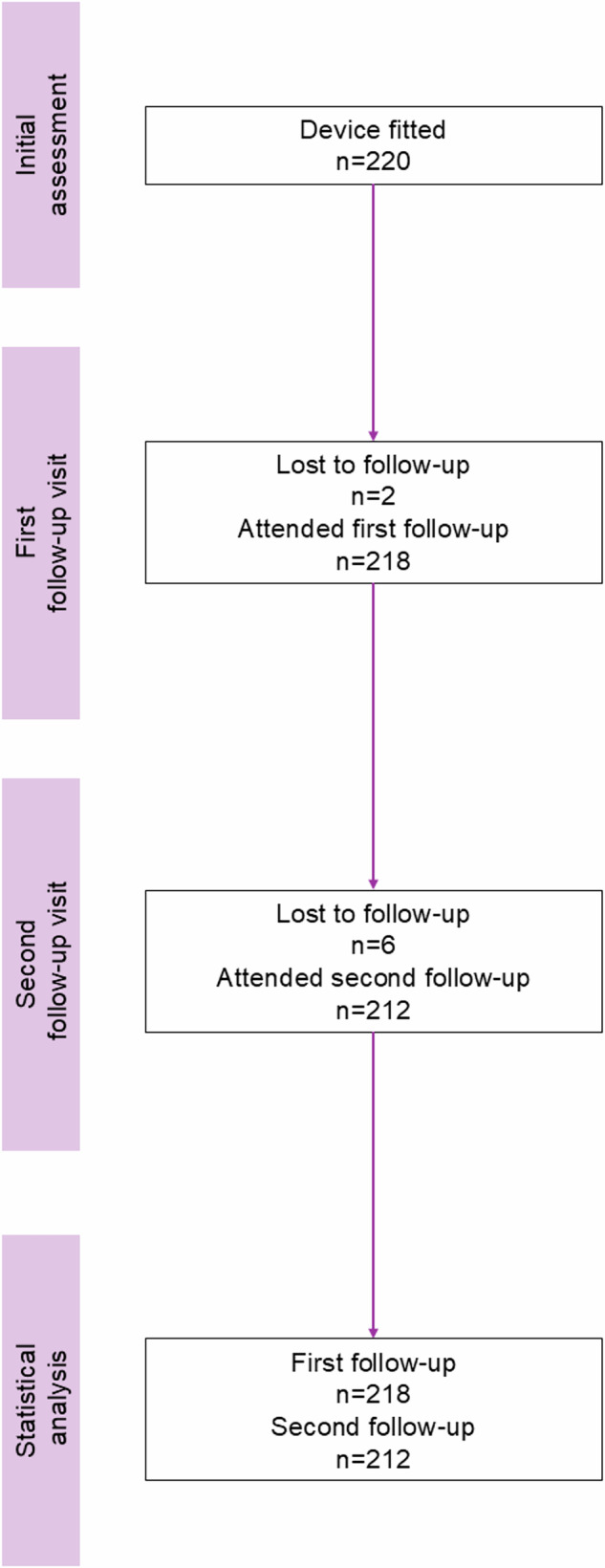
Patient flow diagram. A total of 220 patients with moderate or worse tinnitus severity (THI ≥ 38) were fitted with the Lenire device at initial assessment in accordance with FDA device labeling. *n*: number of participants at each respective phase.

A total of 220 patients satisfied the FDA labeling criterion of a THI score ≥ 38 and were fitted with the Lenire bimodal neuromodulation device at the Alaska Hearing & Tinnitus Center clinics between May 4, 2023 and March 28, 2024 (Fig. 2). The demographic characteristics of these patients are listed in Table 1. The mean age of patients was 60.3 ± 12.6 years with a mean tinnitus duration of 8.5 ± 10.0 years (median of 5.0 with a range of 0.5–60 years). Of the 220 patients, 73.2% were males and 26.8% were females. The higher proportion of male versus female patients observed in this clinical sample is interesting and may reflect the demographic profile of the Alaska Hearing & Tinnitus Center’s patient base. The patient base could be influenced by factors such as regional healthcare-seeking behaviors or a higher prevalence of tinnitus among males in the specific geographic areas serviced by the Alaska Hearing & Tinnitus Center. It should be noted that the high percentage of males in the current study is consistent with findings from a review conducted by Henry et al. (2005), which analyzed data from the Oregon Tinnitus Data Archive. Their study revealed that males comprised more than double the proportion of the patient-seeking population compared to females^22^. It has also been found that males are more likely than females to discuss tinnitus with a healthcare provider^23^ and that there are gender-associated differences related to tinnitus symptoms and treatment response profiles^24^. Also, the gender difference observed in our study is consistent with findings from previous Lenire clinical trials, where males made up 65.0% of clinical trial participants in TENT-A1, 67.0% in TENT-A2, and 68.8% in TENT-A3^12–14^.

**Table 1:** Demographics and tinnitus characteristics of all available patient data (*N* = 220) at initial assessment

All available patients (*N* = 220)	
*Age (years)*	
Mean ± SD (*N*)	60.3 ± 12.6 (220)
*Sex [% (n/N)]*	
Male	73.2% (161/220)
Female	26.8% (59/220)
*Tinnitus duration at initial assessment (years)*	
Mean ± SD (*N*)	8.5 ± 10.0 (220)
*THI at initial assessment (points)*	
Mean ± SD (*N*)	60.0 ± 17.4 (220)
*Hearing loss at initial assessment (dB HL)*	
Mean ± SD (*N*)	
Right ear	19.6 ± 12.4 (215)
Left ear	20.4 ± 13.5 (214)

Mean hearing loss at the initial assessment was calculated using the average of 500, 1000, and 2000 Hz for each ear.

*THI* Tinnitus Handicap Inventory, *dB HL* decibel hearing level, *N* total number of participants in this study with available data, *n* number of participants in a specific subgroup with available data.

At FU1, only two patients were lost to follow-up, and six more patients were lost to follow-up at FU2 (Fig. 2), resulting in a high retention rate of 96.4%. There were consistent characteristics of patients who were lost to follow-up relative to the full cohort (see Supplementary Table 1). For analysis, there were data for 218 patients at FU1 and 212 patients at FU2.

### Clinical efficacy and safety of bimodal treatment are replicable in the real world

**Fig. 3:**
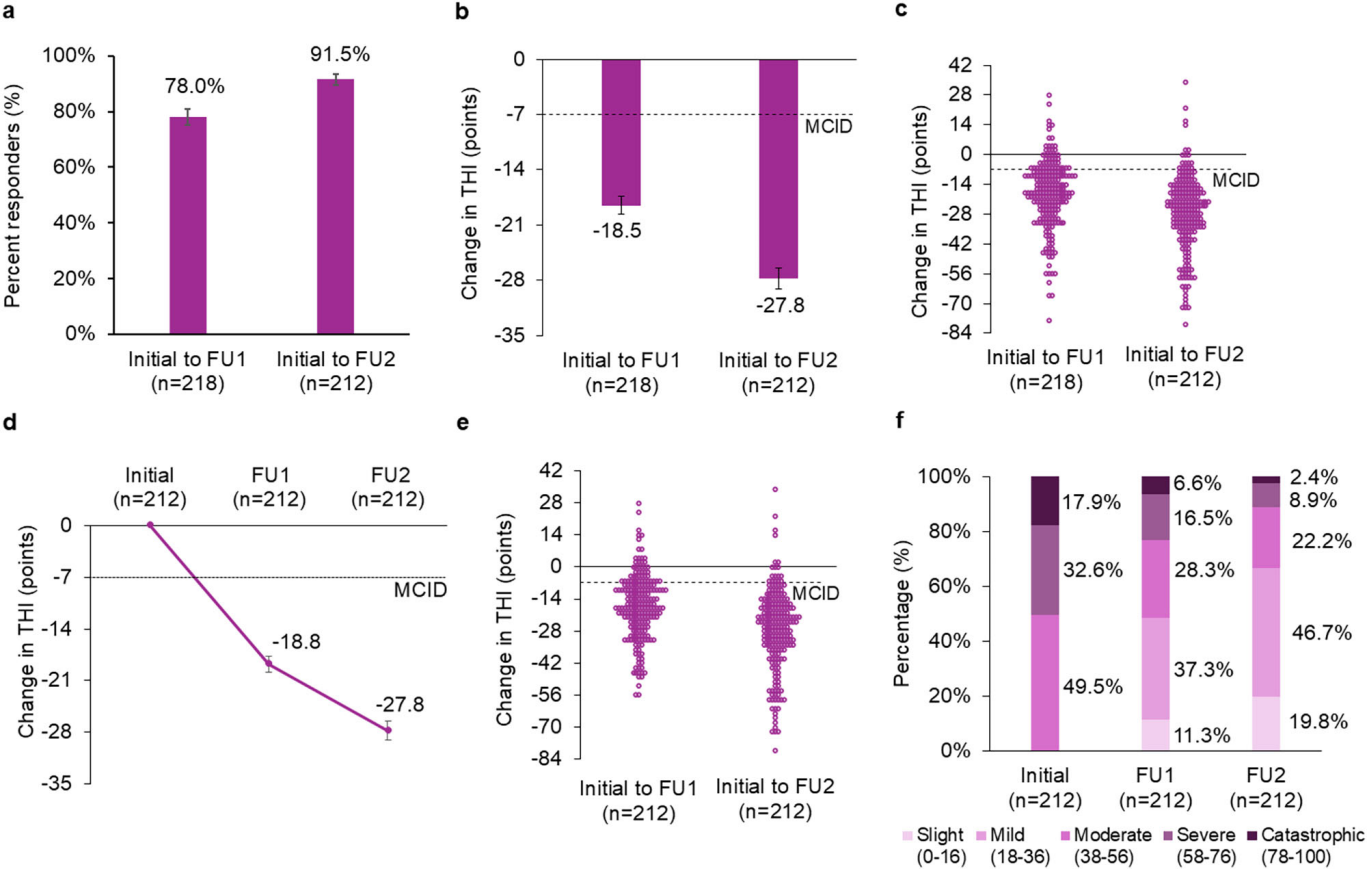
Analyses of Tinnitus Handicap Inventory (THI) change from initial assessment to first follow-up (FU1) and second follow-up (FU2). **a** Percent responders (MCID improvement in THI > 7 points) from initial assessment to FU1 (*n* = 218) and FU2 (*n* = 212); Standard error of mean (SEM) bars are shown. Two-sided *Z*-test of proportions for comparison between groups; *p* = 0.0001. **b** Mean improvement in THI score from initial assessment to FU1 (*n* = 218) and FU2 (*n* = 212); SEM bars are shown. Independent *t*-test for comparison between groups; *p* = 0.00001. **c** Change in THI for each patient from initial assessment to FU1 (*n* = 218) and FU2 (*n* = 212). **d** Mean change in THI from initial assessment to FU1 and FU2 for patients who returned at both follow-up visits (*n* = 212); SEM bars are shown. Paired *t*-test for comparison between groups from initial assessment to FU1 versus to FU2; *p* = 0.00001. **e** Change in THI for each patient from initial assessment to FU1 and FU2 for patients who returned at both follow-up visits (*n* = 212). **f** Percentage of patients (*n* = 212) in the various THI severity categories at initial assessment, FU1, and FU2. The THI severity is categorized as slight (0–16 points), mild (18–36 points), moderate (38–56 points), severe (58–76 points), and catastrophic (78–100 points). MCID minimal clinically important difference, THI Tinnitus Handicap Inventory. *n* number of participant data points represented in each respective bar or plot.

Confirming the benefit of the Lenire treatment for tinnitus observed in previous large-scale clinical studies^12–14^, our primary endpoint analysis demonstrated that 91.5% (95% CI: 86.9%, 94.5 %) of patients with moderate or worse tinnitus severity achieved a clinically significant benefit exceeding the MCID after approximately 12 weeks of treatment (Fig. 3a), corresponding to 27.8 ± 1.3 (mean ± SEM) points reduction in tinnitus severity (Fig. 3b, c). Encouragingly, even after ~6 weeks of treatment by FU1 (i.e., only halfway through the recommended treatment plan), 78.0% (95% CI: 72.0%, 83.0%) of patients already achieved a clinically significant benefit (Fig. 3a), corresponding to 18.5 ± 1.1 (mean ± SEM) points reduction in tinnitus severity (Fig. 3b, c). For completeness, the mean changes in THI from initial assessment to FU1 and FU2 for patients who returned at both follow-up visits are shown in Fig. 3d, e, depicting additional improvement in tinnitus symptoms over time with continued treatment. As shown across different types of analyses in Fig. 3, there is a significant improvement in tinnitus symptoms over time achieved with continued use of the Lenire treatment after adjusting for multiple comparisons. Clinical efficacy results in terms of responder rate and mean changes in THI are consistently observed for male and female participants (Supplementary Fig. 1), with responses for individual patients shown as scatter plots in Supplementary Fig. 2.

As the THI scores can be categorized into various levels of severity^25^, it can also be insightful for data interpretation to plot how severity categories change after tinnitus treatment^26^. In Fig. 3f, among patients who returned for both follow-up visits, the severity distribution at the initial assessment was 49.5% in the ‘Moderate’ group, 32.6% in the ‘Severe’ group, and 17.9% in the ‘Catastrophic’ group. By FU2, these proportions had substantially decreased to 22.2% in the ‘Moderate’ group, 8.9% in the ‘Severe’ group, 2.4% in the ‘Catastrophic’ group, in which 66.5% of patients moved into the ‘Slight’ and ‘Mild’ groups.

Across 220 patients, only eight were lost to follow-up by FU2. Of the six of the eight patients who did return for FU1 (but not FU2), five of the six already had improvements in THI scores after 6 weeks of treatment (see Supplementary Table 2), supporting the notion that patients who are lost to follow-up can be responders to treatment. Even assuming the worst-case scenario that all eight of those lost to follow-up were non-responders by FU2, the responder rate would still be high at 88.2% (194 out of 220).

In terms of safety, there have been no device-related serious adverse events (SAEs) or medical field experience events that warranted reporting to the manufacturer or FDA that were outside the normal issues experienced at the Alaska Hearing & Tinnitus Center during the standard care of patients with tinnitus. There were four patients who experienced noticeable increases in their THI scores and reported not benefiting from the treatment at the 12-week assessment. These patients received additional support, including CBT, counseling, breathing exercises, and other habituation methods, to help manage their tinnitus. Of these four patients, all but one have successfully been supported in managing their tinnitus. The remaining patient is currently facing a tragic personal and health event unrelated to Lenire's treatment, which is likely contributing to their elevated tinnitus status. It is important to note that all patients at the Alaska Hearing & Tinnitus Center are provided education and counseling for their tinnitus; however, patients who experience a persistent increase in tinnitus are supported by a strict protocol at the clinic that was created in line with formal CBT approaches. Overall, the Lenire treatment has continued to exhibit a high benefit to safety profile as observed in the FDA pivotal trial^12^.

### High self-reported benefit rate is consistent with 7-point MCID for THI

**Fig. 4:**
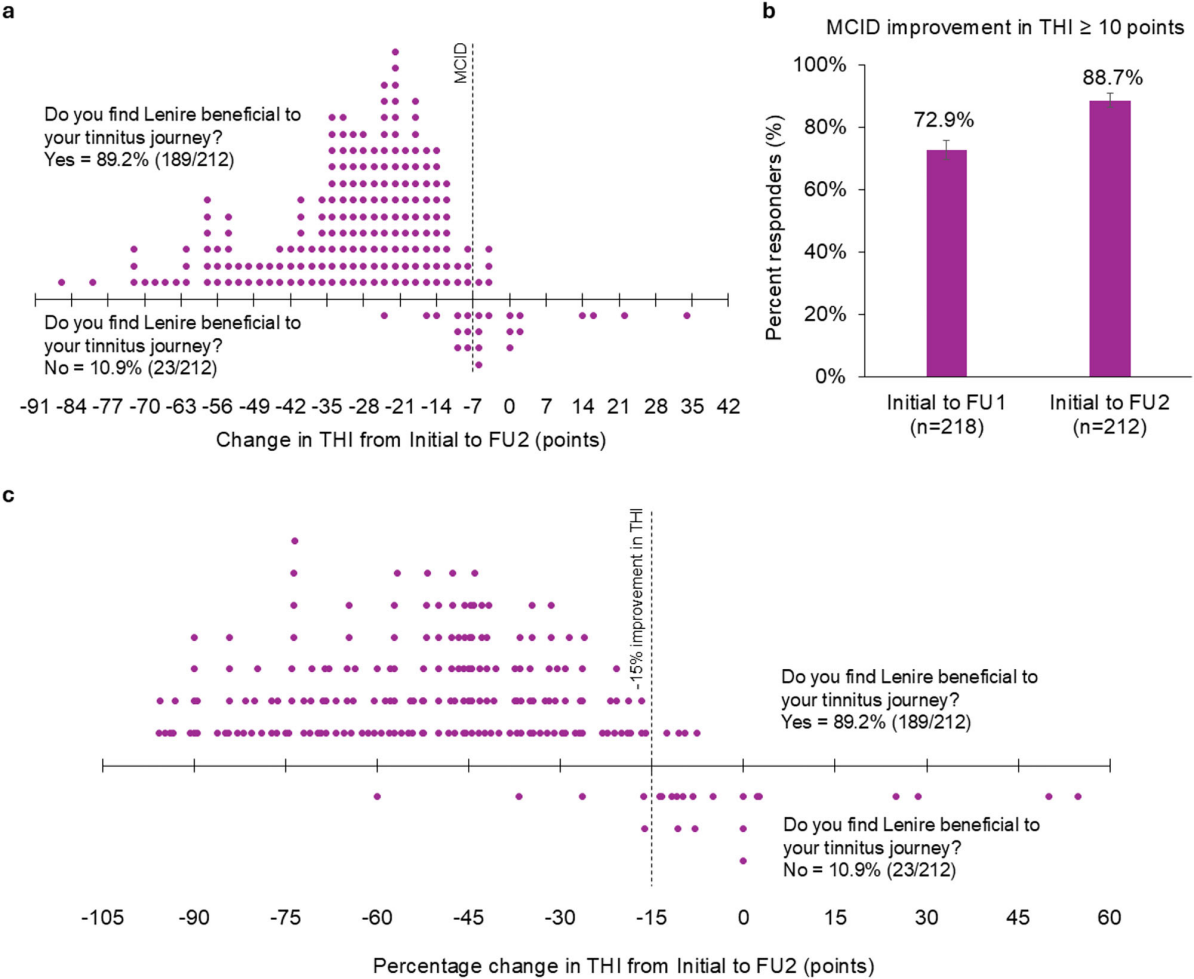
Patient satisfaction rate with the Lenire treatment device. **a** Patient reported the benefit of using the Lenire device for treating tinnitus in relation to their improvement in THI score, demonstrating that the benefit is well aligned with an MCID cut-off of seven points. **b** Percent responders (SEM) from initial assessment to FU1 and FU2 using a more conservative MCID of 9 points (i.e., greater than or equal to 10 points) where there is a more equivalent number of misses versus false hits; i.e., in **a**, if using an MCID of 9 points, there are seven patients with less than clinically meaningful improvement in tinnitus severity but high satisfaction in the upper-right quadrant (misses) versus six patients with clinically meaningful improvement but low satisfaction in the lower-left quadrant (false hits). **c** This plot is similar to the plot in **a** except abscissa is now the percentage change in THI score from initial assessment to FU2 and the cut-off for a clinically meaningful improvement in tinnitus is now set at a ≥15% decrease in THI. Using this cut-off, we can see that patient-reported benefit still aligns well with the THI change. MCID minimal clinically important difference, FU1 first follow-up visit, FU2 second follow-up visit. *n* number of participant data points represented in each respective bar.

At FU2, we directly asked patients if they found Lenire to be beneficial for their tinnitus journey; a high percentage (89.2%) of patients indicated yes (Fig. 4a). It is noteworthy that the patients who reported that they benefitted from Lenire were aligned well with those who improved by at least the MCID cut-off of 7 points in their THI score (Fig. 4a), consistent with previous research that defined the MCID for THI^15^. At a more conservative cut-off of 9 points (i.e., THI ≥ 10), there would be a more equivalent number of patients who benefitted from treatment that would be at or below that cut-off (seven missed responders) versus those who did not benefit who would be at or above that cut-off (six false responders). This conservative MCID of 9 points still leads to a high responder rate of 88.7% (95% CI; 83.7%, 92.3 %) by FU2 (Fig. 4b). Therefore, an MCID of 7 points is a clinically consistent criterion for representing a minimal meaningful benefit in tinnitus symptoms, with an upper conservative criterion of 9 points, and is based on real-world evidence with a large patient cohort treated with Lenire.

There have been recent recommendations for using a percentage change (i.e., 15%^27^) instead of an absolute MCID criterion of 7 points. Figure 4c presents a similar plot to that shown in Fig. 4a, except the abscissa is plotted in terms of percentage change in THI score from initial assessment to FU2, demonstrating that a 15% cut-off also aligns well with patients reporting a benefit from Lenire for their tinnitus journey, where 89.6% (190 of 212) achieved an improvement beyond 15% with an average improvement in THI of 46.8% at FU2 across patients (i.e., about half reduction in tinnitus symptom severity).

## Discussion

The main objective of this retrospective chart review was to confirm the clinical efficacy and safety of the Lenire bimodal treatment in a real-world clinical setting in the United States, since obtaining FDA approval for the treatment in March 2023. The indications for use for Lenire are to alleviate symptoms of tinnitus in patients 18 years of age and older suffering from at least moderate severity tinnitus (THI ≥ 38 at initial assessment). In this patient population, the results were positive and consistent with previous large-scale clinical trials and initial real-world evidence published from Europe^12–14,28^, demonstrating that 91.5% of patients exceeded the MCID after approximately 12 weeks of treatment. In a worst-case scenario using a more conservative MCID of 9 points, there was still a high responder rate of 88.7%; or even assuming the eight lost to follow-up patients were all non-responders (out of 220 patients), then the responder rate would still be at 88.2%. Directly asking patients if they benefitted from the Lenire treatment also led to a high success rate of 89.2%. In terms of safety, there were no serious or major device-related adverse events reportable to the manufacturer or the FDA from the Alaska Hearing & Tinnitus Center. Overall, as observed in the FDA pivotal trial and previous large-scale clinical studies^12–14^, the real-world clinical data supports a high benefit-to-risk profile for the Lenire treatment.

Encouragingly, the proportion of patients who benefitted from treatment was well aligned with those who received a clinically meaningful improvement from the Lenire intervention as assessed with the validated THI^15^. An MCID of 7 points for THI has been demonstrated to be a valid clinically relevant criterion for assessing meaningful benefit from an intervention, based on our real-world clinical data with the Lenire treatment in a large patient cohort. Furthermore, a 15% cut-off for percentage improvement in THI also aligned well with those who reported benefits in their tinnitus from Lenire treatment.

Another objective of this study was to evaluate the feasibility and effectiveness of a hybrid approach to tinnitus management using the Lenire treatment, which integrates both in-person and telehealth services. Telehealth encompasses different modalities and is used for diverse health conditions and patient populations^29^. In tinnitus, to decrease the burden of repeated in-person care, treatment delivered via telehealth is on the rise^17^. Since March 2020, associated with the COVID pandemic, both hybrid and fully remote telehealth services for patients with tinnitus have become more mainstream with benefits shown throughout the therapeutic process^17^. However, it has been highlighted in a systematic review of telehealth interventions for tinnitus (iCBT, internet-based interventions, self-help devices, and smartphone apps) that the main barriers to success are due to a high dropout rate and lack of adherence to treatment^17^. Encouragingly, our high responder rates along with the high satisfaction and retention rates indicate that patients had a positive and effective experience with our hybrid delivery model of tinnitus care for bimodal treatment that incorporates telehealth and in-person services. In our experience, a hybrid approach allows for more personalized, patient-centered care while maximizing the use of technology to enhance ongoing treatment and monitoring. In this study, only seven patients (3.2%) opted for in-clinic follow-up appointments. Changes in THI scores for those who completed in-person follow-up assessments are documented in Supplementary Table 3. The high percentage of patients who selected virtual follow-up assessment (96.8% of 218 at FU1 and 96.7% of 212 at FU2), along with high satisfaction and retention rates, demonstrates that patients were willing and had a positive experience with the hybrid delivery model of tinnitus care. This hybrid model holds promise for expanding access to effective tinnitus treatment to a much larger and more diverse patient population in an accessible and scalable way.

Aligned with the objective to assess the feasibility of incorporating telehealth into the Lenire tinnitus treatment pathway, it is important to mention the practicalities of adding the device to an already established tinnitus clinic. As is the case with any tinnitus treatment, the time allocated per patient is dependent on how disturbed a patient is by their tinnitus symptoms, and some patients may require additional support. The Alaska Hearing & Tinnitus Center has added the Lenire device to its standard treatment pathway. The Lenire-specific visits, including fitting, take up to two hours of clinician time over the course of the first three months of treatment. This is the typical amount of time that a clinician would need to treat a patient with a hearing aid. The new pathway has been easily implemented in the clinic and has allowed patients to take back control of their tinnitus treatment as they can attend follow-up visits and complete treatment from the comfort of their own homes through virtual visits.

One of the limitations of this study is the absence of patient device usage data, as the device needed to be connected to a laptop in the clinic for data extraction, whereas follow-up assessments were conducted remotely. Compliance data has been collected in previous Lenire clinical trials, demonstrating high compliance rates across three clinical trials (82–84%^12–14^; where minimum treatment compliance was defined as at least 18 or 36 h of use across a 6- or 12-week period, respectively). High satisfaction (89.2%) and retention rates (96.4%) were reported in the current study, supporting a likelihood of high compliance similar to previous Lenire studies. However, the possibility that patients used the device for longer than prescribed in the current study, potentially contributing to a higher responder rate than that observed in the Lenire clinical trials, cannot be ruled out.

Tinnitus is a well-known heterogenous disorder that requires experienced clinicians with a deep knowledge of methods and technologies available to help the patient manage their disturbing condition. To the best of our knowledge, these results represent the first real-world evidence for Lenire tinnitus treatment published from a United States cohort and provide confirmation that the treatment efficacy of the Lenire device can be successfully translated to and replicated in a clinical practice setting. When new treatment devices enter clinics and patient care settings, the benefits can be lower than observed in structured clinical studies due to a greater diversity of patients and variability in clinical processes in the real-world environment. Therefore, it is important to continue tracking the effectiveness of novel treatments in the real-world setting to build evidence-based care pathways for patients with tinnitus. Our patient results combined with previous clinical studies^12–14^ further support the Lenire device as an effective and safe treatment for tinnitus.

## Supplementary information

Supplementary Information

Description of Additional Supplementary Files

Supplementary Data

Reporting summary

## Data Availability

Source data related to Figs. 3,  4, Supplementary Figs. 1 and  2 are in the document named Supplementary Data. Access to the raw individual-level data may be obtained, contingent on appropriate ethics approval and data-sharing agreements, by contacting EEM (clinicaldataqueries@neuromoddevics.com) for the purposes of confirming the analysis in the paper. Responses to valid requests will be reasonably attempted and initiated within 10 working days of receipt beginning 3 months and ending 5 years after this article publication.
