# Supplementary material for: Retrospective chart review demonstrating effectiveness of bimodal neuromodulation for tinnitus treatment in a clinical setting
Source: Commun Med (Lond). 2025 Apr 28;5:112. doi: 10.1038/s43856-025-00837-3 (PMC12037789; doi:10.1038/s43856-025-00837-3)
Supplement: Supplementary file 1 — Supplementary Information [file 43856_2025_837_MOESM1_ESM.pdf]

Supplementary Information

Retrospective chart review demonstrating effectiveness of bimodal neuromodulation for tinnitus treatment in a clinical setting

Emily E. McMahan, Hubert H. Lim

Supplementary Table 1. Demographics and tinnitus characteristics for patients who did not attend their second follow-up (FU2) visit.

Supplementary Figure 1: Mean improvement in Tinnitus Handicap Inventory (THI) score from initial assessment to first follow-up (FU1) and second follow-up (FU2) by sex.

Supplementary Figure 2. Scatter plots of Tinnitus Handicap Inventory (THI) scores of each patient at initial assessment versus first follow-up (FU1), and initial assessment versus second follow-up (FU2).

Supplementary Table 2. Tinnitus Handicap Inventory (THI) scores at initial assessment and first follow-up (FU1) visit for patients who did not attend their second follow-up (FU2) visit.

Supplementary Table 3. Change in Tinnitus Handicap Inventory (THI) for patients who attended in-person clinic visits from initial assessment to first follow-up (FU1) and initial assessment to second follow-up (FU2).

Supplementary Methods. Alaska Hearing & Tinnitus Center Chart Review for Hybrid Care of Tinnitus with Bimodal Neuromodulation -Research Proposal.

Supplementary Table 1. Demographics and tinnitus characteristics for patients who did not attend their second follow-up (FU2) visit.

	Age (year range)	Sex	THI at initial assessment	Tinnitus duration at initial assessment	Mean hearing loss at initial assessment (dB HL)
Patient 1	40–44	Male	76	23	Right ear, 3.3<br>Left ear, 1.7
Patient 2	50–54	Male	76	5	Right ear, 5<br>Left ear, 1.7
Patient 3	55–59	Male	84	1	Right ear, 6.6<br>Left ear, 8.3
Patient 4	55–59	Female	74	5	Right ear, 10<br>Left ear, 13
Patient 5	45–49	Male	54	6	Right ear, 15<br>Left ear, 16.7
Patient 6	65–69	Male	40	3	Right ear, 20<br>Left ear, 20
Patient 7	55–59	Male	76	20	Right ear, 42<br>Left ear, 30
Patient 8	60–64	Male	82	10	Right ear, 15<br>Left ear, 62

Mean hearing loss at initial assessment was calculated using the average of 500 Hz, 1000 Hz and 2000 Hz for each ear. THI: Tinnitus Handicap Inventory; dB HL: decibel hearing level.

Supplementary Figure 1: Mean improvement in Tinnitus Handicap Inventory (THI) score from initial assessment to first follow-up (FU1) and second follow-up (FU2) by sex.

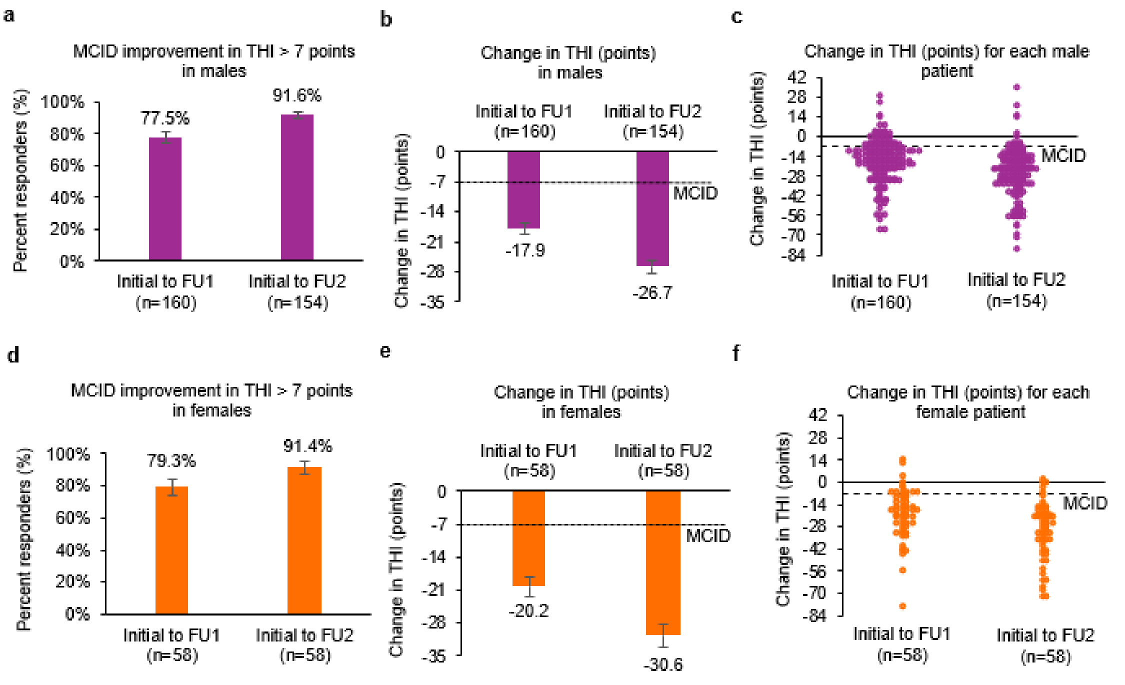

(a) In males: percent responders (Minimal Clinically Important Different, MCID improvement in THI > 7 points) from initial assessment to FU1 and FU2; Standard Error of Mean (SEM) bars are shown. Two-sided Z-test of proportions for comparison between groups, initial to FU1 (n=160) and initial to FU2 (n=154); p=0.0005**.** **(b)** In males: mean improvement in THI score from initial assessment to FU1 and FU2; SEM bars are shown. Independent t-test for comparison between groups, initial to FU1 (n=160) and initial to FU2 (n=154); p=0.00001. **(c)** Data for change in THI for each male patient from initial assessment to FU1 and FU2. **(d)** In females: percent responders (MCID improvement in THI > 7 points) from initial assessment to FU1 and FU2; SEM bars are shown. Two-sided Z-test of proportions for comparison within groups, initial to FU1 (n=58) and initial to FU2 (n=58); p=0.0014**.** **(e)** In females: mean improvement in THI score from initial assessment to FU1 and FU2; SEM bars are shown. One sample t-test for comparison between groups, initial to FU1 (n=58) and initial to FU2 (n=58); p=0.00001. **(f)** Data for change in THI for each female patient from initial assessment to FU1 and FU2. All data supports a significant improvement in tinnitus symptoms over time with continued use of the Lenire treatment that is consistently observed across males and females. n = number of participant data points represented in each respective bar.

Supplementary Figure 2. Scatter plots of Tinnitus Handicap Inventory (THI) scores of each patient at initial assessment versus first follow-up (FU1), and initial assessment versus second follow-up (FU2).

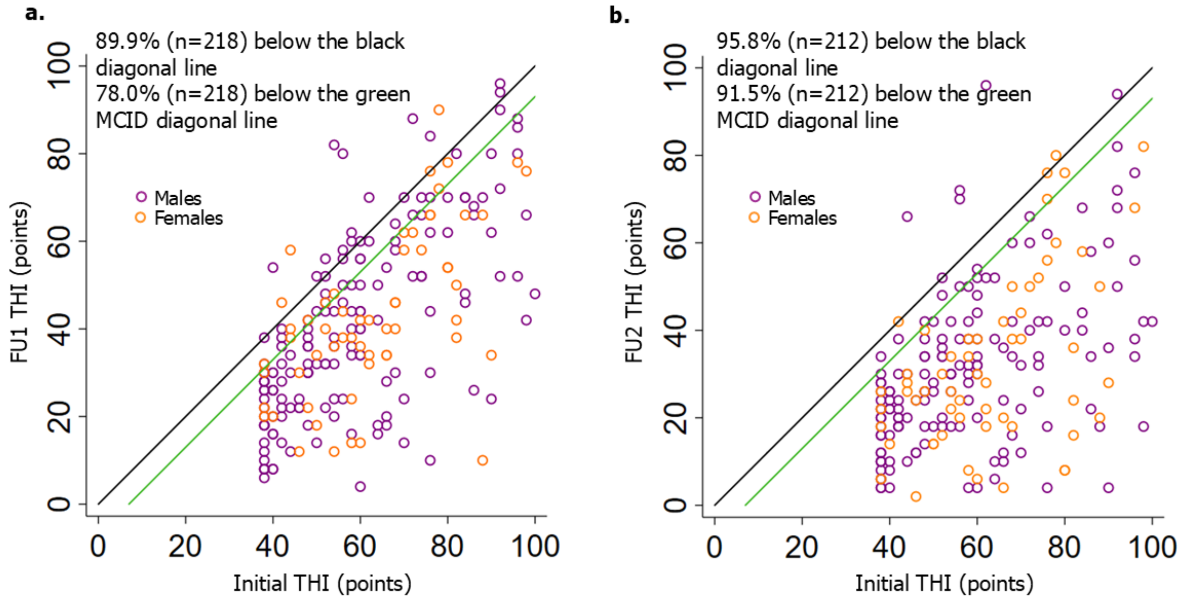

Scatter plots of THI scores of each patient at **(a)** initial assessment versus FU1, and **(b)** initial assessment versus FU2. Each circle representing each patient that is below the green line corresponds to an improvement by at least seven points on the THI scale based on the MCID (Minimal Clinically Important Different), whereas each circle below the black line corresponds to any improvement in THI score. n = number of participant data points represented in each respective scatter plot.

Supplementary Table 2. Tinnitus Handicap Inventory (THI) scores at initial assessment and first follow-up (FU1) visit for patients who did not attend their second follow-up (FU2) visit.

	**THI at initial assessment**	**THI at FU1**	**Change in THI score from initial assessment to FU1**
Patient 1	76		
Patient 2	76	44	-32
Patient 3	84	70	-14
Patient 4	74		
Patient 5	54	44	-10
Patient 6	40	54	14
Patient 7	76	70	-6
Patient 8	82	80	-2

Responder to treatment (Minimal Clinically Important Different, MCID ≥ 7 points on the THI).

Negative points equate to a reduction in THI score.

Supplementary Table 3. Change in Tinnitus Handicap Inventory (THI) for patients who attended in-person clinic visits from initial assessment to first follow-up (FU1) and initial assessment to second follow-up (FU2).

	**THI at initial assessment**	**Change in THI from initial assessment to FU1**	**Change in THI from initial assessment to FU2**
Patient 1	70	-8	-26
Patient 2	76	-66	-72
Patient 3	58	-20	-24
Patient 4	38	0	-4
Patient 5	38	-32	-34
Patient 6	82	-40	-58
Patient 7	52	-30	-32

Responder to treatment (Minimal Clinically Important Different, MCID ≥ 7 points on the THI). Negative points equate to a reduction in THI score, in which six out of seven patients exhibited a clinically significant improvement substantially beyond the MCID.

Supplementary Methods

Alaska Hearing & Tinnitus Center Chart Review for Hybrid Care of Tinnitus with Bimodal Neuromodulation - Research Proposal
Version: 1.0
Date: 19/03/2024

This proposal is being submitted for an exempt research review. It is believed that the research described below falls under 45 CFR 46.104(d)(4) iii which states that:

- Under the revised rule a secondary review of data may be eligible for determination of exempt status if the data
  - (iii) involves only information collection and analysis involving the investigator's use of identifiable health information when that use is regulated under 45 CFR parts 160 and 164, subparts A and E, for the purposes of "health care operations" or "research (i.e. the data is Protected Health Information (PHI) and otherwise meets the technical, administrative and physical safeguards in the HIPAA standards [45 CFR parts 160 and 164])
- Exempt Category 4 at 45CFR46.104(d)(4) expressly states consent is not required if the study involves secondary research use of identifiable private information or identifiable biospecimens and meets one of the above the criteria for exemption.
- All patients proposed to be included in this analysis have consented to a HIPAA Waiver which includes the disclosure of data for research purposes once done so lawfully.

Protocol Title: Alaska Hearing & Tinnitus Center Chart Review for Hybrid Care of Tinnitus with Bimodal Neuromodulation

Principle Investigator: Emily E. McMahan, Au.D

Location of secondary data (charts) to be reviewed:

Alaska Hearing & Tinnitus Center, [redacted]

Alaska Hearing & Tinnitus Center, [redacted]

Are all investigators members of the workforce of the facilities/covered entities where the data will be abstracted/reviewed?

Yes [X] **No** [ ] If no, explain how authority to access data will be requested and what agreements (if applicable) will be executed: **N/A**

1. Introduction

This chart review is a retrospective data analysis of real-world data to assess the performance of bimodal neuromodulation when delivered as a hybrid approach in the real-world. At the Alaska Hearing & Tinnitus Center clinics, in-person and telehealth services are used for initial audiological and tinnitus assessments. Fitting of the bimodal neuromodulation device, Lenire® is completed at a subsequent in-person visit. Virtual video calls via

CounselEAR's telehealth portal are primarily used for follow-up assessments, additional consultations, counselling, and education.

Lenire is an FDA approved non-invasive, self-administered, bimodal neuromodulation device intended to provide treatment to reduce the symptoms of tinnitus. The device consists of a Tonguetip®, an intraoral device designed to sit comfortably in the mouth and deliver a gentle electrical stimulation on the tongue's surface to trigger nerves; Bluetooth headphones that plays personalized sounds to the ear, aimed at stimulating the auditory nerve; and a handheld controller which enables patients to adjust the duration and intensity of the treatment. The patient has limited control of the sound volume and tongue stimulation with the handheld controller for ease of comfort and as a safety precaution.

Lenire delivers sound stimulation from the headphones while providing gentle tongue stimulation. The sounds and pulses work together to provide a unique sensory experience that helps to re-train the brain and reduce the perception of tinnitus.

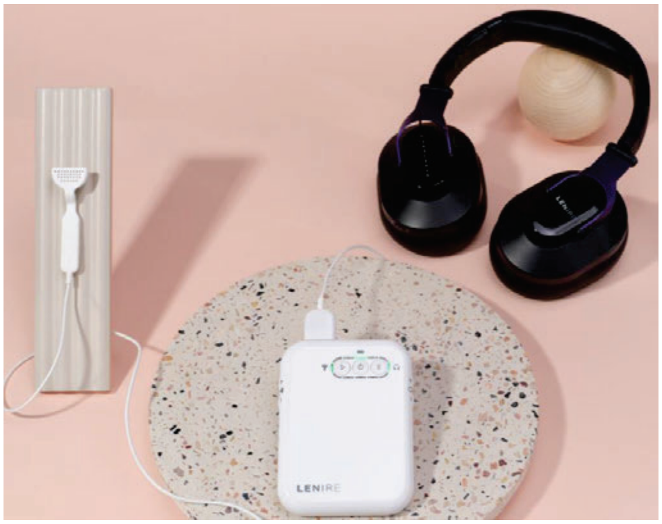

Figure 1: Lenire Device – Headphones, Tonguetip and Controller

In this study, audiological, medical and device records held by Alaska Hearing & Tinnitus Center will be extracted and consolidated to generate one separate 'Chart Review Database'. All patients who are fitted with the Lenire device at the clinic since May 2023 to the date of ethics approval will be included in the analyses. The process of extracting, merging, and validating the databases will be carried out by the clinic personnel who have access to identified data as part of the clinic's day-to-day practice. The final 'Chart Review Database' will be de-identified before analysis.

This database will be used to review the performance of the device and the feasibility of a hybrid tinnitus management approach for bimodal neuromodulation in a real-world clinical setting. Tinnitus management conventionally involves in-person care, however since the COVID-19 pandemic, telehealth has become instrumental for routine follow-ups and consultation. The importance of the discoveries of this study is twofold, to bridge the evidence gap regarding efficiency between clinical research and practice, and to assess if telehealth is a viable option for follow-up assessment as part of the Lenire device standard of care. The Lenire device has only been in the USA market since May 2023, and results from this study can potentially offer insights to future Lenire healthcare providers, either via presentation of the data at conferences or in the form of a peer-reviewed publication.

2. Background

Tinnitus, the perception of phantom sound is a challenging disorder with limited effective therapies [1]. In the USA, tinnitus affects approximately 11.2% of the adult population [2]. Tinnitus is a symptom of varied pathologies. However, the most common risk factor for tinnitus is sensorineural hearing loss [3-5]. One widely held hypothesis is that reduced sensorineural input due to damage of the hair cells or fibers of the auditory nerve causes compensatory changes in firing activity in multiple regions along the ascending auditory and non-auditory pathways that can lead to the manifestation of tinnitus [6-8].

2.1 Previous Treatment Options for Tinnitus

Guidelines on the management and treatment of tinnitus have been published by the American Academy of Otolaryngology—Head and Neck Surgery Foundation (AAO-HNSF) [9], a multidisciplinary European team [10], and the National Institute for Health and Care Excellence in the UK [11].

Evidence of sufficient quality to support a recommendation is only available for cognitive behavioral therapy (CBT). Additionally, sound therapy is described by AAO-HNSF as an option. The published guidelines consistently recommend against pharmacological approaches (e.g., betahistine), nutritional supplements (e.g., ginkgo biloba), and transcranial stimulation due to either insufficient evidence to recommend, or because there is evidence to recommend against.

CBT is intended to help tinnitus patients understand their tinnitus and deal with the associated symptoms, resulting in improvements to their quality of life. Although the effectiveness of CBT has been demonstrated in randomized controlled trials, an extended treatment period (8 months) is required to achieve a significant improvement in tinnitus [12]. Also, the need for specialized, trained therapists to provide CBT is challenging in terms of scalability to a large population of tinnitus patients.

The more scalable and commonly prescribed tinnitus treatment is auditory stimulation, such as sound amplification (e.g., hearing aids), sound therapy (e.g., tinnitus maskers) or a combination of both which incorporates a tinnitus masker into a hearing aid. A Cochrane meta-analysis of six clinical investigations of sound therapy in 2012 found no evidence of a reduction in tinnitus severity or tinnitus loudness compared to interventions such as patient education or relaxation techniques [13]. As a result, there are no formal clinical

recommendations for their singular use in the treatment of tinnitus [14]. Nevertheless, it is generally accepted in the hearing and tinnitus fields that sound therapy can be clinically effective for some tinnitus patients or can drive partial perturbation/improvements in tinnitus symptoms for many individuals (though not clinically significant) [15].

2.2 Telehealth in tinnitus

Remote health services have been proven to be a viable treatment delivery method to improve patients' access to high-quality and cost-effective care [16]. Telehealth encompasses different modalities and is used for diverse health conditions and patient populations [16]. In chronic tinnitus, to decrease the burden of repeated in-person care, treatment delivered via telehealth are on the rise [17]. Since March 2020, both hybrid and fully remote telehealth services for tinnitus patients have become more mainstream with benefits shown throughout the therapeutic process [17].

A current systematic review of telehealth interventions (iCBT, internet-based interventions, self-help devices, and smartphone apps) reported that there is low to moderate quality evidence for the effectiveness of these interventions in reducing tinnitus severity and distress [17]. It was highlighted that the barriers to the success of telehealth are due to a high dropout rate and lack of adherence to treatment [17]. To our knowledge this is the first chart review to evaluate the use of a hybrid tinnitus care model for bimodal neuromodulation in the real world.

The Alaska Hearing & Tinnitus Center applies a hybrid delivery model of tinnitus care incorporating telehealth and in-person services. After an initial consultation (in-person or via telehealth), if a patient is prescribed Lenire an in-person device fitting is completed, and follow-on care is generally completed via telehealth with the option for in-person care for those who are located closer to clinics. Our online services facilitate continued care by providing the possibility for remote tinnitus counselling, education, and additional consultations. In order to better understand and validate bimodal neuromodulation with a hybrid standard of care procedure, we believe it is important to assess our patients' experience and contribute sufficient published data to adequately support tinnitus treatment guidelines in the future.

2.2 What is Bimodal Neuromodulation for Tinnitus

Based on extensive research in animals and several human studies, bimodal neuromodulation using auditory stimulation combined with electrical stimulation of other non-auditory nerves such as the vagus nerve, trigeminal nerve, and somatosensory nerves (e.g., nerves innervating different body regions) has emerged as a promising approach [18-26]. Somatosensory and/or trigeminal inputs can access or modulate neurons throughout the auditory pathway [23, 27-36]. Electrical stimulation of the trigeminal or somatosensory nerves can also activate the ascending reticular activating system of the brain, which consists of neurons involved with emotional, attentional, and cognitive functions [37]. Bimodal stimulation combining electrical stimulation of the trigeminal or somatosensory nerves with sound stimulation can modulate tinnitus-related neuronal activity and potentially drive long-lasting plasticity changes in the brain [38, 39], due to the convergence of inputs into these different auditory and non-auditory regions that can contribute to neural plasticity relevant for tinnitus treatment.

Furthermore, such bimodal stimulation approaches can be delivered via portable medical devices suitable for safe and convenient use by patients in the comfort of their home.

2.3 Previous Investigations on Lenire

Two large-scale clinical trials (TENT-A1 [39] investigation; clinicaltrials.gov: NCT02669069, TENT-A2 [38] investigation; clinicaltrials.gov: NCT03530306) have been published supporting the safety and efficacy of Lenire's tinnitus treatment and for the proposed mechanism of action. These large-scale trials have been peer-reviewed and accepted by highly regarded scientific journals.

TENT-A1 the first large-scale clinical trial, was in 2020 regarded as the largest medical device clinical trials in the tinnitus field [39]. The TENT-A1 investigation reported that bimodal neuromodulation provided clinical benefit for about two-thirds of patients during the 12-week treatment period and achieved significant reductions in tinnitus symptoms that lasted for 12 months after the treatment ended [39]. TENT-A2 further repeated and confirmed the findings in TENT-A1 in a large number of tinnitus participants, demonstrating the consistent in treatment effects across studies [38]. Importantly, in both TENT-A1 [39] and TENT-A2 [38], there were no treatment-related serious adverse events throughout the investigations and there were high treatment compliance rates and satisfaction rates across investigation patients.

[redacted]

Lenire is the first non-invasive bimodal neuromodulation tinnitus treatment device that has consistently been shown to be safe and effective in relieving tinnitus in three large-scale clinical trials. Beyond clinical trials, an independent real-world evidence study of 20 patients who were treated with the Lenire device at HorSys GmbH supported the efficacy and safety of the device [41].

The current study is important to allow continual assessment of the real-world data now available for Lenire and to determine the replicability of treatment outcomes in the real-world setting outside of the European continent. Results from multiple clinics in the USA from patients with a wide range of medical history and tinnitus characteristics will enrich our understanding of the efficiency of Lenire in both a clinical and telehealth setting. Also, findings from this study will inform future Lenire providers on the feasibility of tinnitus management using a hybrid model.

3. Study Design

This is a retrospective chart review of the performance of the Lenire device and the feasibility of the hybrid standard of care procedure for the Lenire device. All adult (18 years and above) patients with subjective tinnitus that have been treated with the Lenire device since May 2023 be included in the analysis. All available electronic medical records collected at the clinic as per standard of care will be accessed, and de-identified to create a 'Chart Review Database' to be used for data analysis consistent with study objectives.

3.1 Study Population

The medical records of adult patients (18 years and older) with subjective tinnitus that have presented at the Alaska Hearing & Tinnitus Center (and associated clinics) for the treatment of their tinnitus and have been prescribed Lenire will be included in the review. [redacted]

3.2 Inclusion Criteria

Tinnitus sufferers of at least 18 years of age who have been prescribed the device.

3.3 Exclusion Criteria

Any patients who meet any of the contraindications of the device are not suitable to be prescribed Lenire.

Lenire is contraindicated:

- For persons who have a pacemaker, defibrillator, or any other active implantable device, unless directed by a physician.
- For persons who are pregnant, unless directed by a physician.
- For persons who have epilepsy or any other condition that may result in loss of consciousness, unless directed by a physician.
- For persons who have any condition that causes impaired sensitivity of the tongue, unless directed by a physician or dentist.
- For persons who have lesions, sores, or inflammation of the oral cavity that could be in contact with the Tonguetip (i.e., tip of tongue and lip area), unless directed by a physician or dentist.
- For persons who have any intermittent or chronic neuralgia in the head and neck area, unless directed by a physician.
- For persons who have tinnitus caused by Meniere's disease, unless directed by a physician. The use of this device has not been evaluated in the TENT clinical studies for Meniere's disease.
- For persons who have tinnitus confirmed to be from an objective source.
- In the presence of oral piercings.

4. Study Objectives and Hypothesis

4.1 Hypothesis

As this is a retrospective chart review analyzing pre-existing data, there are no formal hypothesis testing.

4.2 Primary Objective:

The primary objective for this study is to report on the treatment response of patients using Lenire when delivered using a hybrid standard of care procedure. Treatment outcomes are assessed at the clinic using validated measures such as the Tinnitus Handicap Inventory (THI) [42, 43].

4.3 Additional Objectives:

1. Evaluate treatment responses of patients during treatment compared to the natural changes in severity of tinnitus during waiting time from requesting an appointment to initial assessment.
2. Investigate any correlations between treatment effect and patient demographics/medical history (e.g. age, tinnitus severity, tinnitus duration, use of previous tinnitus treatments).
3. Assess patients' satisfaction and benefit. Usage compliance of the Lenire device may also be assessed.
4. Compare results of the real-world data analysis with that of other available real-world data to show consistent results across clinical settings.

4.4 Primary Endpoint

Mean changes and percentage of patients with a reduction in THI of 7 points or more (minimum clinical important difference (MCID) for THI [43]) from initiation to completion of active tinnitus treatment will be reported.

4.5 Additional Endpoints

Changes in THI scores during the waiting period from request for assessment to initial assessment will be compared to changes in THI scores from initial assessment to post-treatment. Correlations and trends for changes in THI scores in patients with different demographics and tinnitus characteristics will be examined. Rates of satisfaction, and benefit of the Lenire device will be compared to published literature.

Comparisons of response rate and mean changes in subgroups will be completed using the THI severity categories (i.e., none/slight, mild, moderate, severe, and catastrophic groups) and sub-domains of validated THI questionnaire.

5. Study Methods

This study is a retrospective data analysis of real-world clinical data collected as part of standard of care for patients undergoing treatment with Lenire at the Alaska Hearing & Tinnitus Center clinics. Medical records stored in CounselEar Office Management Solutions (OMS) will be accessed, merged into a 'Chart Review Database' and exported as an excel spreadsheet (.csv). All investigators (i.e., clinicians/research associates) at *Alaska Hearing & Tinnitus Center* have access to the CounselEar OMS using secure access credentials. For this retrospective analysis,

a study database will be generated by investigator(s) who have access to the records as part of their standard duties at the clinic. In the EDC, a unique identifier is used to link data between visits for the same patient. Compliance data, initial request data and audiological data may be merged into the 'Chart Review Database' from their respective sources. Upon exporting the data no identifiable information will be retained in the final study database nor will there be a need to re-identify the data after the study database has been finalized. Patients will not be contacted and have consented to a HIPAA Waiver Notice ('Notice of Privacy Practices') prior to initiating treatment at the clinic which allows for data to be used for research purposes. It was verbally explained during the initial consultation how the data would be used to further research on the Lenire device given the Alaska Hearing & Tinnitus Center was included in the first limited commercial roll-out of the device and included as a Phase I provider. [redacted] A template of the clinic's Privacy Policy and Notice of Privacy Practices will be attached to the Institutional Review Board (IRB) submission as supporting information.

[redacted]

5.1 Data Records Review and Identification

1. **Identifiers recorded for this study:** All identifiers will be removed from the finalized version of the study database once the export has been validated and any merging of initial request or compliance data completed.
2. **Source of records to be reviewed:** Medical records held by the Alaska Hearing & Tinnitus Center clinics on the EDC, device compliance data stored on the laptop used to fit the device and accessed from the fitting software of the device.
3. **Describe how the charts to be reviewed will be identified:** The clinic will search for patients fitted with a Lenire device in their records and extract the full list of patients and assessments required as part of this review. Data from these patients will then be used to link the data from the other sources listed above.
4. **Describe who will identify charts to be reviewed:** Investigator(s) at the clinic that have access to the records as part of their standard duties at the clinic will identify the records to be reviewed.
5. **Describe the process for recording data from those eligible:** Data will be exported directly from the identified medical files into a spreadsheet that can be downloaded as a .csv file. This will then be cross-checked for accuracy that all relevant patients are included, and all assessments have exported correctly. Data may also be exported from the device fitting software and merged with the medical record .csv file using patient identifiers such as name or medical number. Data of patients who request an initial appointment by filling out an online request form may be exported and merged with the medical record

.csv file using patient identifiers (i.e., email, contact number). All personal identifiers will be deleted from the dataset once it is finalized.

6. **Describe the process for de-identification:** When the final study database is ready for analysis all patient identifiers such as name, contact details and medical number will be permanently deleted from the excel file prior to saving the file for analysis. No version of the .csv file will be maintained with the patient identifying information.
7. **Describe the process for re-identification:** A unique identifier will be used to link data between sheets in the database or between visits, but this identifier will not be retained to re-identify patients nor will it be possible to use it to link back to the source records. Data will only be presented in publications and presentations in aggregate or de-identified form in order to ensure re-identification is not possible outside of the clinic.

6. Data collection

Routine access of medical records by the study team which they have access to as part of their day-to-day role in the clinic. Medical records include clinical data from assessment request forms, questionnaires, medical and tinnitus history, device logs and audiological assessments. The original purpose of collecting the data was for the routine clinical care and assessment of a patient with tinnitus. The data is originally collected with identifiers that are required to contact the patient as part of the clinic's routine operations. Once the data is extracted from the medical records, identifiers will be removed prior to the analysis process. Data will be reported in aggregate and anonymized form so that individual subjects will not be identified through the presentation of results. For the research project, the investigator will not need to re-contact or re-identify patients for any reason in the future. The original medical records are still maintained by the investigator for ongoing care of patients. See Appendix A for details on the data collected. Questionnaires, tinnitus and audiological assessments and device usage data are the main forms of data collected.

7. Data Confidentiality and Data Security

The following details how data will be stored to safe-guard confidentiality:

1. The final study database will be stored in a de-identified manner. It will be stored on a secure network with firewall protection. Data is only accessible from password protected and encrypted laptops and by those involved in the study. Only electronic data is captured at the clinic and utilized for the purposes of this study.
2. The PI, investigators/clinicians at the Alaska Hearing & Tinnitus Center clinics and clinicians/research associates required to analyze the data will have access to the data. Personnel that have access to the identifiable data are those that have authorized access for their standard duties at the clinic.
3. For compliance with HIPAA regulations, investigators will retain copies of any consents to the Notice of Privacy Practices for a minimum of 7 years after the authorization or alteration was last obtained or in effect. The study data will also be maintained for a minimum of 7 years in line with this. All data is stored electronically via CounselEar's cloud-based storage and will be destroyed via the deletion of all files and

data back-ups at the appropriate time.

8. Risks and Benefits

8.1 Risks

As patients have already undergone treatment and the data has been collected already the only risk to be considered for the completion of this retrospective chart review is a breach of confidentiality, this is typically the sole risk associated with retrospective studies. Appropriate technical and organizational measures are already in place at the clinic to ensure the confidentiality of the collection and storage of patients' medical records are maintained in compliance with HIPAA regulations, irrespective of the database they are held in. Additional risk is associated with the export and analysis of the study database; however, these risks are minimized by storing the data on secure servers (firewall and password protected) and the removal of all identifiers prior to analysis and so the risk of a breach of confidentiality is considered to be low.

8.2 Benefits

The subjects whose charts are reviewed are not likely to receive any benefit from the proposed research; however, society (such as future patients and other healthcare providers) and investigators will benefit from the knowledge gained. To ensure widespread benefit within the science and clinical communities the findings will be disseminated at conferences and through peer-reviewed journals in de-identified, aggregate form.

9. Data management and Statistical Considerations

9.1 Statistical Considerations

The purpose of the retrospective chart review is to examine the listed research questions on readily accessible existing data collected at the Alaska Hearing & Tinnitus Center clinics. Considering that the dataset described in this study contains detailed records of tinnitus patients, it has the potential to help further understand the benefits of the Lenire treatment, monitor and improve clinical practice. Therefore, the analyses will be partly exploratory, conducted to identify statistical patterns, correlations, and trends to direct specific hypothesis generation.

The de-identified database will be analyzed by a researcher with the relevant statistical skills. Support from an experienced biostatistician may be required, if this is the case then the necessary data sharing agreements will be put in place and only the completely de-identified data shared unless the necessary agreements authorize access to the full dataset.

At a high level, exploratory data analysis will be used to understand the contents of the dataset to formulate specific questions and to prepare for more advanced statistical modeling. Exploratory data analysis techniques will be used to visualize and summarize descriptive statistics. Demographic and tinnitus characteristics of patients will be summarized using measures such as central tendency (mean, median), dispersion (range, standard error,

standard deviation) and skewness of the population (percentile, kurtosis). Results will be presented in tables or visualized using histograms, boxplots or scatter plots and will be discussed in relation to other tinnitus populations in the literature to establish the generalizability of patients seeking treatment with Lenire.

Based on results of data exploration, inferential statistics may be used to compare means of variables of interest (e.g., t-test for THI before and after treatment). Point estimates (e.g., means), and interval estimates (e.g., confidence interval) will be reported. Findings will be presented and discussed in relation to results obtained in previous clinical investigations of the device to confirm the performance of the device in the real-world in relation to previous data.

If required, correlation tests and regressions may be performed to demonstrate the extent to which variables are related. All assumptions of regression models, linearity, normality, multicollinearity, and homoscedasticity will be assessed and if assumptions are violated, data transformation and other appropriate statistical methodology will be applied. Results from regression models will enable us to determine reasons and potential confounders for treatment outcomes (e.g., reasons for success or failure of treatment). This will enable the identification of contraindications and any risk of using the device.

All statistical analyses will be carried out on the complete case database. Where data is missing, different sensitivity analyses may be explored if there is evidence that unobserved values are not missing-at-random.

Cut-offs for clinically meaningful changes for the various assessments from initial to follow-up will be determined in accordance with values from validated research literature (e.g., a seven point or more reduction in THI after treatment is considered a clinically meaningful change).

All analyses will be carried out using STATA, R, SPSS, or other relevant statistical programs.

9.2 Data Quality

Data will be exported from the patients' medical files, the device fitting software, audiology software, and assessment request forms and collated into one study database using a common identifier such as Patient File # or Patient ID. Once the data is collated, the data will be cross-referenced against the source to ensure that no errors have occurred through the process of exporting and merging the dataset. Once the dataset has been completed, the data will be de-identified and stored in a secure folder on the clinic's system (e.g. HIPAA compliant Google Drive, part of the patient management suites). No version of the study database with identifying data will be kept – all previous versions of the exported and collated database with identifying data will be destroyed from local and cloud servers.

9.3 Data Storage and Transmission

The review of medical records will be carried out by the investigators who are employees of the Alaska Hearing & Tinnitus Center clinics, who, in the course of their duties at the clinic, would ordinarily have access to the personal data of individuals held by the clinic that was obtained for the provision of health care to the patients. The PI and clinicians/research associates are the primary individuals collating the data from databases at the clinic

into a study database. All data are stored on password protected and encrypted computers. The investigators are trained in HIPAA compliance and have authorized access to the data at the clinic. No identifiable data will be transferred from the covered entity (unless lawfully allowed e.g. as per HIPAA waiver or Business Associate Agreement) or stored in the final version of the study database.

10. HIPAA Authorization

All patients being treated at the Alaska Hearing & Tinnitus Center clinics e-sign consent to the clinic's 'Notice of Privacy Practices' this agreement includes the sharing of medical records for the purposes of research. The notice describes how medical information may be used or disclosed at the practice and how a patient can access this information. A blank version of this document is submitted as part of the supporting documentation to the IRB.

It is believed that in the proposed study that the use or disclosure of protected health information in the current research involves no more than minimal risk to privacy of individuals, based on the presence of the following elements:

- As previously mentioned, all identifiers will be removed from the final study database. No personnel who are not authorized to have access to personal data as part of their daily duties, or as part of the HIPAA waiver, will be given access to the study database until identifiers have been removed. The study database will not be disclosed to anyone outside of the clinic's normal operations which have been disclosed as part of the Notice of Privacy Practices which the patient has consented to prior to initiating treatment at the clinic. Only the minimum amount of data required to assess the objectives of this project will be exported and included in the study database. Data to be included is listed in Appendix A.
- Identifiers will be deleted at the earliest opportunity, i.e. once the study database is validated and finalized. After which there is no requirement to be able to re-identify the database nor will patients need to be contacted in regards to the study database.
- This study involves reviewing questionnaires and health information related to tinnitus and audiological health. The only risk to this study is confidentiality risk, which is minimized through using standard HIPAA provision and data security. All Alaska Hearing & Tinnitus Center clinics HIPAA policies will be followed. Study documents are saved on password protected drives on firewall protected servers, no identifiable data will be transferred from the covered entity (unless lawfully allowed as part of the healthcare operations of the clinic) and access is restricted to personnel working at the clinic that have authorized access to the data as part of their standard duties, or as per the HIPAA waiver. The data will only be used for the purposes set forth in this protocol unless otherwise permitted under the Notice of Privacy Practices.

References

1. Davis, A. and A. El Refaie, *Epidemiology of Tinnitus. Tinnitus Handbook (Singular Audiology Text), Singular Pub.* Group, 2000.

2. Batts, S. and K.M. Stankovic, *Tinnitus prevalence, associated characteristics, and related healthcare use in the United States: a population-level analysis.* The Lancet Regional Health–Americas, 2024. **29**.
3. Møller, A.R., et al., *Textbook of tinnitus*. 2010: Springer Science & Business Media.
4. Rauschecker, J.P., A.M. Leaver, and M. Mühlau, *Tuning out the noise: limbic-auditory interactions in tinnitus.* Neuron, 2010. **66**(6): p. 819-826.
5. Schaette, R., *Tinnitus in men, mice (as well as other rodents), and machines.* Hearing Research, 2014. **311**: p. 63-71.
6. De Ridder, D., et al., *Phantom percepts: tinnitus and pain as persisting aversive memory networks.* Proceedings of the National Academy of Sciences, 2011. **108**(20): p. 8075-8080.
7. De Ridder, D., et al., *An integrative model of auditory phantom perception: tinnitus as a unified percept of interacting separable subnetworks.* Neuroscience & Biobehavioral Reviews, 2014. **44**: p. 16-32.
8. Meyers, E.C., et al., *Enhancing plasticity in central networks improves motor and sensory recovery after nerve damage.* Nature communications, 2019. **10**(1): p. 5782.
9. Tunkel, D.E., et al., *Clinical practice guideline: tinnitus.* Otolaryngology–Head and Neck Surgery, 2014. **151**(2_suppl): p. S1-S40.
10. Cima, R., et al., *A multidisciplinary European guideline for tinnitus: diagnostics, assessment, and treatment.* Hno, 2019. **67**(1): p. 10-42.
11. Lewis, S., et al., *Assessment and management of tinnitus: Summary of NICE guidance.* BMJ, 2020. **368**.
12. Cima, R.F., et al., *Specialised treatment based on cognitive behaviour therapy versus usual care for tinnitus: a randomised controlled trial.* Lancet, 2012. **379**(9830): p. 1951-9.
13. Hobson, J., E. Chisholm, and A. El Refaie, *Sound therapy (masking) in the management of tinnitus in adults.* Cochrane Database of Systematic Reviews, 2012(11).
14. Fuller, T.E., et al., *Different teams, same conclusions? A systematic review of existing clinical guidelines for the assessment and treatment of tinnitus in adults.* Frontiers in psychology, 2017. **8**: p. 206.
15. Henry, J.A., et al., *Reevaluating the use of sound therapy for tinnitus management: Perspectives on relevant systematic reviews.* Journal of Speech, Language, and Hearing Research, 2022. **65**(6): p. 2327-2342.
16. Snoswell, C.L., et al., *The clinical effectiveness of telehealth: a systematic review of meta-analyses from 2010 to 2019.* Journal of telemedicine and telecare, 2023. **29**(9): p. 669-684.
17. Demoen, S., et al., *Effectiveness of Telerehabilitation Interventions for Self-management of Tinnitus: Systematic Review.* Journal of Medical Internet Research, 2023. **25**: p. e39076.
18. De Ridder, D., et al., *Safety and efficacy of vagus nerve stimulation paired with tones for the treatment of tinnitus: a case series.* Neuromodulation: Technology at the Neural Interface, 2014. **17**(2): p. 170-179.
19. Engineer, N.D., et al., *Reversing pathological neural activity using targeted plasticity.* Nature, 2011. **470**(7332): p. 101-104.
20. Hamilton, C., et al., *An investigation of feasibility and safety of bi-modal stimulation for the treatment of tinnitus: an open-label pilot study.* Neuromodulation: Technology at the Neural Interface, 2016. **19**(8): p. 832-837.
21. Koehler, S.D. and S.E. Shore, *Stimulus-timing dependent multisensory plasticity in the guinea pig dorsal cochlear nucleus.* PloS one, 2013. **8**(3): p. e59828.
22. Ma, X. and N. Suga, *Augmentation of plasticity of the central auditory system by the basal forebrain and/or somatosensory cortex.* Journal of Neurophysiology, 2003. **89**(1): p. 90-103.
23. Markovitz, C.D., et al., *Investigating a new neuromodulation treatment for brain disorders using synchronized activation of multimodal pathways.* Scientific reports, 2015. **5**(1): p. 1-12.
24. Marks, K.L., et al., *Auditory-somatosensory bimodal stimulation desynchronizes brain circuitry to reduce tinnitus in guinea pigs and humans.* Science Translational Medicine, 2018. **10**(422): p. eaal3175.

25. Offutt, S.J., et al., *Suppression and facilitation of auditory neurons through coordinated acoustic and midbrain stimulation: investigating a deep brain stimulator for tinnitus.* Journal of neural engineering, 2014. **11**(6): p. 066001.
26. Weinberger, N.M., *Associative representational plasticity in the auditory cortex: a synthesis of two disciplines.* Learning & memory, 2007. **14**(1-2): p. 1-16.
27. Aitkin, L., C. Kenyon, and P. Philpott, *The representation of the auditory and somatosensory systems in the external nucleus of the cat inferior colliculus.* Journal of Comparative Neurology, 1981. **196**(1): p. 25-40.
28. Gloeckner, C.D., et al. *A new concept for noninvasive tinnitus treatment utilizing multimodal pathways*. in *2013 35th Annual International Conference of the IEEE Engineering in Medicine and Biology Society (EMBC)*. 2013. IEEE.
29. Gruters, K.G. and J.M. Groh, *Sounds and beyond: multisensory and other non-auditory signals in the inferior colliculus.* Frontiers in neural circuits, 2012. **6**: p. 96.
30. Itoh, K., et al., *Direct projections from the dorsal column nuclei and the spinal trigeminal nuclei to the cochlear nuclei in the cat.* Brain Res, 1987. **400**(1): p. 145-50.
31. Ledoux, J.E., et al., *Topographic organization of convergent projections to the thalamus from the inferior colliculus and spinal cord in the rat.* Journal of Comparative Neurology, 1987. **264**(1): p. 123-146.
32. Levine, R.A., *Somatic (craniocervical) tinnitus and the dorsal cochlear nucleus hypothesis.* American journal of otolaryngology, 1999. **20**(6): p. 351-362.
33. Levine, R.A., et al., *Evidence for a tinnitus subgroup responsive to somatosensory based treatment modalities.* Progress in brain research, 2007. **166**: p. 195-207.
34. Robards, M.J., *Somatic neurons in the brainstem and neocortex projecting to the external nucleus of the inferior colliculus: an anatomical study in the opossum.* Journal of Comparative Neurology, 1979. **184**(3): p. 547-565.
35. Shore, S.E., L.E. Roberts, and B. Langguth, *Maladaptive plasticity in tinnitus—triggers, mechanisms and treatment.* Nature Reviews Neurology, 2016. **12**(3): p. 150-160.
36. Vanneste, S., et al., *Transcutaneous electrical nerve stimulation (TENS) of upper cervical nerve (C2) for the treatment of somatic tinnitus.* Experimental brain research, 2010. **204**: p. 283-287.
37. De Cicco, V., et al., *Trigeminal, visceral and vestibular inputs may improve cognitive functions by acting through the locus coeruleus and the ascending reticular activating system: a new hypothesis.* Frontiers in Neuroanatomy, 2018: p. 130.
38. Conlon, B., et al., *Different bimodal neuromodulation settings reduce tinnitus symptoms in a large randomized trial.* Scientific reports, 2022. **12**(1): p. 10845.
39. Conlon, B., et al., *Bimodal neuromodulation combining sound and tongue stimulation reduces tinnitus symptoms in a large randomized clinical study.* Science Translational Medicine, 2020. **12**(564): p. eabb2830.
40. Network, M.D., *US FDA Grants De Novo Approval to Neuromod's Lenire Device for Tinnnitus.* 2023.
41. Buechner, A., et al., *Real-world clinical experience with bimodal neuromodulation for the treatment of tinnitus-A case series.* Brain Stimulation: Basic, Translational, and Clinical Research in Neuromodulation, 2022. **15**(2): p. 383-384.
42. Kleinstäuber, M., I. Frank, and C. Weise, *A confirmatory factor analytic validation of the Tinnitus Handicap Inventory.* Journal of Psychosomatic Research, 2015. **78**(3): p. 277-284.
43. Zeman, F., et al., *Tinnitus handicap inventory for evaluating treatment effects: which changes are clinically relevant?* Otolaryngology--Head and Neck Surgery, 2011. **145**(2): p. 282-287.

APPENDIX A: DATA COLLECTION

Given that this is a Chart Review, all patient assessments have already been conducted and no additional assessments or procedures will be undertaken. Data was collected at several timepoints including Screening (Request for Assessment), Initial Assessment, Device Fitting Visit and Follow-up Visits.

The following assessments and methods were collected as part of standard of care for the treatment of tinnitus at the Alaska Hearing & Tinnitus Center clinics. These data points are not exhaustive of all data collected at the clinic but provide input for each of the endpoints detailed previously. Only data collected as part of standard of care will be included in the retrospective analysis. Given that the database will be generated from the existing data in the clinic's patient management system, separate case report forms (CRFs) will not be attached for this review.

The following information provides further detail on the standard of care assessments performed and the data that may be included in the final study database.

Medical History

- Tinnitus history: location, duration, previous tinnitus treatments, nature, type, and sound of tinnitus
- Other medical conditions
- Tinnitus etiology
- Concomitant prescription medication
- Demographics (gender, age, race, ethnicity, employment status, current/previous member of military)
- Questions relating to investigation inclusion and exclusion criteria

Updated medical history is captured at each visit, including changes in tinnitus characteristics or any noticeable differences in hearing.

Tinnitus Handicap Inventory (THI)

The THI is a validated psychometric questionnaire used to determine the severity of tinnitus symptoms and is one of the most widely established instruments for assessing tinnitus symptom severity [42, 43]. The reliability of the THI has previously been demonstrated [43], and it has been validated in many languages [42].

The THI is comprised of 25 questions, which can be answered as "No," "Yes," or "Sometimes," to which a numerical score of 0, 4, or 2 is assigned, respectively. The total THI score is the sum of all item scores and can range from 0 ("no handicap") to 100 ("catastrophic handicap"). The minimal clinically important difference (MCID) reported for THI is 7 points [43] and represents a clinically meaningful change in tinnitus symptoms. A THI questionnaire will be completed by the patient at each visit.

Lenire Device

- Device Fitting: Patient's pure-tone audiometric (PTA) thresholds (250 Hz to 8 kHz) will be measured at the initial assessment visit and subsequently used to configure the sound stimuli to just above their hearing threshold at each tone frequency. Electrical stimulation intensity is configured for each patient by adjusting the intensity from sub-threshold to supra-threshold sensations to a comfortable intensity across different electrodes.
- Compliance: The Lenire device records the times and duration of use to an inbuilt memory chip. When the patient returns their device, this usage data is extracted. This data will provide information regarding device usage and compliance with treatment plan.
- Device Traceability: Each device has a serial number which is recorded in the log files. In the event of an issue with the device, the investigational device would be returned to the manufacturer according to standard site and device manufacturer safety reporting procedures.
- Stimulation Parameter Sets: The stimulation parameter setting is set for each patient, as per the Clinician's Manual (IFU-0023) and Lenire User Manual (IFU-0022). A record of the parameter settings will be included in the database.

Pure Tone Audiometry

Air-conduction Pure Tone Audiometry (PTA) may be conducted binaurally to assess patients' hearing thresholds pre- and post- treatment in accordance with standard practice. Measurements may be made at the standard audiometric test frequencies {250, 500, 1k, 2k, 3k, 4k, 6k and 8k Hz using standard audiometry equipment.

Tympanometry

Tympanometry is an objective test that measures the movement and function of the middle ear and eardrum (tympanic membrane). The equipment being used is maintained as per internal procedures.

Otoscopy

Otoscopy is a clinical procedure used to examine structures of the ear, particularly the external auditory canal, tympanic membrane, and middle ear.

Satisfaction Questions

Participants will be asked to complete two questions to provide their feedback on their overall benefit and satisfaction of the treatment at the FINAL visit.
