# Supplementary material for: Retrospective chart review demonstrating effectiveness of bimodal neuromodulation for tinnitus treatment in a clinical setting
Source: Commun Med (Lond). 2025 Apr 28;5:112. doi: 10.1038/s43856-025-00837-3 (PMC12037789; doi:10.1038/s43856-025-00837-3)
Supplement: Supplementary file 2 — Description of Additional Supplementary Files [file 43856_2025_837_MOESM2_ESM.pdf]

Description of Additional Supplementary Files

File name: Supplementary Data
Description: Source data related to Fig. 3, Fig. 4, Supplementary Fig. 1, and Supplementary Fig. 2
