# Supplementary material for: Retrospective chart review demonstrating effectiveness of bimodal neuromodulation for tinnitus treatment in a clinical setting
Source: Commun Med (Lond). 2025 Apr 28;5:112. doi: 10.1038/s43856-025-00837-3 (PMC12037789; doi:10.1038/s43856-025-00837-3)
Supplement: Supplementary file 4 — Reporting summary [file 43856_2025_837_MOESM4_ESM.pdf]

nature portfolio

Corresponding author(s): Hubert H. Lim

Last updated by author(s): 13/3/25

Reporting Summary

Nature Portfolio wishes to improve the reproducibility of the work that we publish. This form provides structure for consistency and transparency in reporting. For further information on Nature Portfolio policies, see our Editorial Policies and the Editorial Policy Checklist.

Statistics

For all statistical analyses, confirm that the following items are present in the figure legend, table legend, main text, or Methods section.

n/a	Confirmed	
☐	☒	The exact sample size (*n*) for each experimental group/condition, given as a discrete number and unit of measurement
☐	☒	A statement on whether measurements were taken from distinct samples or whether the same sample was measured repeatedly
☐	☒	The statistical test(s) used AND whether they are one- or two-sided<br>*Only common tests should be described solely by name; describe more complex techniques in the Methods section.*
☒	☐	A description of all covariates tested
☐	☒	A description of any assumptions or corrections, such as tests of normality and adjustment for multiple comparisons
☐	☒	A full description of the statistical parameters including central tendency (e.g. means) or other basic estimates (e.g. regression coefficient) AND variation (e.g. standard deviation) or associated estimates of uncertainty (e.g. confidence intervals)
☐	☒	For null hypothesis testing, the test statistic (e.g. *F*, *t*, *r*) with confidence intervals, effect sizes, degrees of freedom and *P* value noted<br>*Give P values as exact values whenever suitable.*
☒	☐	For Bayesian analysis, information on the choice of priors and Markov chain Monte Carlo settings
☒	☐	For hierarchical and complex designs, identification of the appropriate level for tests and full reporting of outcomes
☒	☐	Estimates of effect sizes (e.g. Cohen's *d*, Pearson's *r*), indicating how they were calculated

Our web collection on statistics for biologists contains articles on many of the points above.

Software and code

Policy information about availability of computer code

Data collection	Data were collected using CounselEar Office Management Solution (OMS) maintained by clinicians at Alaska Hearing & Tinnitus Center clinics.
Data analysis	Standard statistical methods in STATA version 15 and were plotted/presented with Excel version 2403.

For manuscripts utilizing custom algorithms or software that are central to the research but not yet described in published literature, software must be made available to editors and reviewers. We strongly encourage code deposition in a community repository (e.g. GitHub). See the Nature Portfolio guidelines for submitting code & software for further information.

Data

Policy information about availability of data

All manuscripts must include a data availability statement. This statement should provide the following information, where applicable:

- Accession codes, unique identifiers, or web links for publicly available datasets
- A description of any restrictions on data availability
- For clinical datasets or third party data, please ensure that the statement adheres to our policy

Source data related to Fig.3, Fig.4, Supplementary Fig.1, and Supplementary Fig.2 are in the document named Supplementary Data. Access to the raw individual level data may be obtained, contingent on appropriate ethics approval and data sharing agreements, by contacting EEM (clinicaldataqueries@neuromoddevics.com) for the purposes of confirming the analysis in the paper. Responses to valid requests will be reasonably attempted and initiated within 10 working days of receipt beginning 3 months and ending 5 years after this article publication.

Research involving human participants, their data, or biological material

Policy information about studies with human participants or human data. See also policy information about sex, gender (identity/presentation), and sexual orientation and race, ethnicity and racism.

Reporting on sex and gender	Primary endpoint analyses for responder rates were carried out according to participants' self-reported gender. Of the 220 patients fitted with the bimodal stimulation device, 161 were male and 59 were female.
Reporting on race, ethnicity, or other socially relevant groupings	Race, ethnicity and social groupings are not reported.
Population characteristics	Total number of participants at initial assessment =220. Mean age of the full cohort = 60.3 years. Mean Tinnitus Handicap Inventory (THI) score for the full cohort (n=220) at initial assessment was 60.0 points.
Recruitment	Patients who were fitted with the Lenire device from May 4, 2023 to March 28, 2024. Patients must meet the basis of the inclusion/exclusion criteria stipulated in the protocol. There were no potential self-selection bias or other biases present that the investigators are aware of.
Ethics oversight	This study has been reviewed by an institutional review board (IRB), to ensure patients rights and welfare as a research participant are protected and that the research study is carried out in an ethical manner. The study was reviewed by a registered IRB (name: Advarra IRB; IRB number: 00000971; Study Protocol number: Pro00077817) and was determined to be exempt from IRB oversight based on the Department of Health and Human Services regulations found at 45 CFR 46.104(d)(4). All methods were carried out in accordance with relevant guidelines and regulations.

Note that full information on the approval of the study protocol must also be provided in the manuscript.

Field-specific reporting

Please select the one below that is the best fit for your research. If you are not sure, read the appropriate sections before making your selection.

☒ Life sciences ☐ Behavioural & social sciences ☐ Ecological, evolutionary & environmental sciences

For a reference copy of the document with all sections, see nature.com/documents/nr-reporting-summary-flat.pdf

Life sciences study design

All studies must disclose on these points even when the disclosure is negative.

Sample size	The retrospective chart review has a cohort of 220 patients.
Data exclusions	All available data for the 220 patients were included in the study.
Replication	Replication of the results in this study confirms previously published clinical trials using the Lenire bimodal neuromodulation device.
Randomization	No randomization was possible as the study is a chart review of a cohort of patients who underwent treatment with the Lenire bimodal neuromodulation device in a clinical practice setting.
Blinding	No blinding was possible as the study is a chart review of a cohort of patients who underwent treatment with the Lenire bimodal neuromodulation device in a clinical practice setting.

Reporting for specific materials, systems and methods

We require information from authors about some types of materials, experimental systems and methods used in many studies. Here, indicate whether each material, system or method listed is relevant to your study. If you are not sure if a list item applies to your research, read the appropriate section before selecting a response.

Materials & experimental systems

n/a	Involved in the study
☒	☐ Antibodies
☒	☐ Eukaryotic cell lines
☒	☐ Palaeontology and archaeology
☒	☐ Animals and other organisms
☐	☒ Clinical data
☒	☐ Dual use research of concern
☒	☐ Plants

Methods

n/a	Involved in the study
☒	☐ ChIP-seq
☒	☐ Flow cytometry
☒	☐ MRI-based neuroimaging

Clinical data

Policy information about clinical studies

All manuscripts should comply with the ICMJE guidelines for publication of clinical research and a completed CONSORT checklist must be included with all submissions.

Clinical trial registration: This is a retrospective chart review.

Study protocol: The study protocol is submitted with the manuscript.

Data collection: A total of 220 patients' medical records, health evaluations and tinnitus assessment were retrieved from CounselEAR Office Management Solutions by the clinician at Alaska Hearing & Tinnitus Center clinics.

Outcomes: The primary endpoint is the responder rate after approximately 12 weeks of treatment with bimodal stimulation using the Lenire device. A responder to treatment is defined as an improvement of 7 points or more on the validated Handicap Inventory (THI) scale. Mean changes in THI scores for patients who returned for follow-up visits are also reported. In addition to the THI, patients' respond (Yes or No) to the question 'Do you find Lenire beneficial to your tinnitus journey?' is reported.

Plants

Seed stocks: n/a

Novel plant genotypes: n/a

Authentication: n/a
